# The Effect of Removal of External Proteins PsbO, PsbP and PsbQ on Flash-Induced Molecular Oxygen Evolution and Its Biphasicity in Tobacco PSII

**DOI:** 10.3390/cimb46070428

**Published:** 2024-07-08

**Authors:** Sonia Krysiak, Kvetoslava Burda

**Affiliations:** Faculty of Physics and Applied Computer Science, AGH University of Krakow, al. Mickiewicza 30, 30-059 Krakow, Poland; soniakrysiak@gmail.com

**Keywords:** photosynthesis, Mn_4_CaO_5_ complex—water-oxidizing enzyme, oxygen evolution, extrinsic proteins, photosystem II, higher plant

## Abstract

The oxygen evolution within photosystem II (PSII) is one of the most enigmatic processes occurring in nature. It is suggested that external proteins surrounding the oxygen-evolving complex (OEC) not only stabilize it and provide an appropriate ionic environment but also create water channels, which could be involved in triggering the ingress of water and the removal of O_2_ and protons outside the system. To investigate the influence of these proteins on the rate of oxygen release and the efficiency of OEC function, we developed a measurement protocol for the direct measurement of the kinetics of oxygen release from PSII using a Joliot-type electrode. PSII-enriched tobacco thylakoids were used in the experiments. The results revealed the existence of slow and fast modes of oxygen evolution. This observation is model-independent and requires no specific assumptions about the initial distribution of the OEC states. The gradual removal of exogenous proteins resulted in a slowdown of the rapid phase (~ms) of O_2_ release and its gradual disappearance while the slow phase (~tens of ms) accelerated. The role of external proteins in regulating the biphasicity and efficiency of oxygen release is discussed based on observed phenomena and current knowledge.

## 1. Introduction

**Figure 1:**
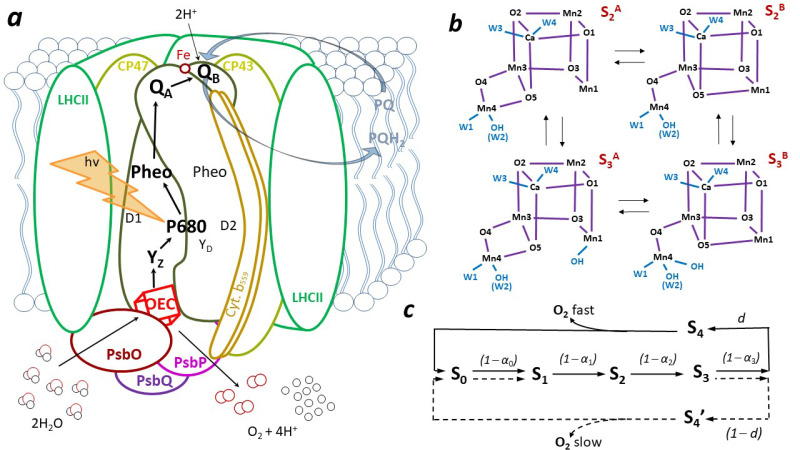
(**a**) Simplified scheme of photosystem II (PSII) for higher plants localized in the inner membranes of chloroplasts, called thylakoids. For clarity, small proteins are omitted except for cytochrome b_559_ [26,27]. Two polypeptides, D1 (PsbA) and D2 (PsbD), form the core of PSII. Two inner antenna subunits, CP43 and CP47, are attached to it. The OEC on the PSII donor side, containing four Mn ions, one Ca ion, and five oxygen atoms forming the Mn_4_CaO_5_ cluster, is protected in higher plants by three outer proteins of about 17 kDa (PsbQ), 23 kDa (PsbP), and 33 kDa (PsbO) [28]. The thick orange lightning shows the excitation of the chlorophyll pair at the reaction center, P680, and the black arrows show the direction of linear electron flow within PSII. (**b**) The currently accepted open (left column) and closed (right column) conformations of the *S*_2_ and *S*_3_ states and their possible transformations upon attaching a water molecule are shown. The water molecules W1 and W2 (here it is deprotonated) are ligands to Mn4, and W3 and W4 are ligands to Ca. The ligand O5 is nominally closest to the entrance of the broad channel (also called Cl1 channel), the ligand O4 to the entrance of the narrow channel (also called O4 channel), and the ligand O1 to the entrance of the *large* channel (also called O1 channel) [29,30,31,32,33,34,35]. The additional water molecule bound to Ca ion during the *S*_2_ → *S*_3_ transition described in [36], which can be a precursor of the O6 atom, is not included here. (**c**) Scheme of the 5S-state model explicitly considering the *S*_4_ state and its longer-lived isomer *S*_4_*_’_*, which are associated with fast (*d*—the probability parameter) and slow ((1 − *d*)—the probability parameter) O_2_ release, respectively [37,38]. The transition probability between *S_i_* → *S_i_*_+1_ states is (1 − *α_i_*), where *α_i_* is the miss parameter.

The appearance of the first photosynthetic organisms capable of extracting electrons and protons from water determined the direction of the evolution of living organisms on Earth. This is the result of a process called oxygenic photosynthesis, which is responsible for increasing and maintaining the amount of O_2_ in the Earth’s atmosphere. Only organisms possessing photosystem II (PSII), including cyanobacteria, algae, and higher plants, are able to oxidize water 2H_2_O → 4e^−^ + 4H^+^+ O_2_↑. PSII is the only photosystem with a high enough redox potential for water oxidation [1,2]. It has a water-oxidizing enzyme known as an oxygen-evolving complex (OEC), which can cyclically accumulate four positive charges, bind water, release electrons and protons, and support the formation of O=O bonds and the release of O_2_ molecules [3,4,5]. The PSII complex and the components involved in the linear electron transport process are shown in Figure 1a. PSII is a light-driven plastoquione oxidoreductase.

A major step forward in understanding the functioning of the OEC was Joliot’s observation of periodic oscillations of oxygen evolution in dark-adapted chloroplasts under the influence of short saturating flashes [3] and the linear four-step model of the oxidation cycle proposed by Kok et al. [4]. The cycling transient states of the OEC are called *S_i_*_(_*_i =_*_ 0__,1,2,3,4__)_ states and are assigned to a particular arrangement and oxidation state of the Mn_4_CaO_5_ complex (subscript indicates the number of accumulated charges, see Figure 1c, Equations (S1)–(S3) in the Supplementary Materials). When sufficient oxidizing power is accumulated, water molecules are split, an O=O bond is formed, and O_2_ is released.

The detected high degree of conformational variability of the individual *S_i_* states may suggest an adaptive ability of the Mn_4_CaO_5_ complex to maintain an optimal level of OEC activity under certain external and internal conditions. Two redox isomers of the *S*_2_ state with distinctive EPR signals were detected [6,7,8,9]. It was shown that the *S*_2_ state may exist in a low spin (LS) state characterized by a multiline signal with g ≈ 2 and in a high spin (HS) state with g ≈ 4. These two states are isoenergetic and can be converted into each other (Figure 1b). The transition between open (denoted A, LS) and closed (denoted B, HS) Mn_4_CaO_5_ structures is associated with changes in the coupling between the Mn ions that form the manganese cluster, resulting in an Mn1(III)Mn4(IV) ↔ Mn1(IV)Mn4(III) electron exchange and O5 ligand displacement [10]. Recently, even two stable transient *S*_2_ closed conformers were suggested [11]. The heterogeneity observed in *S*_2_ may already be the result of two different intermediate states *S*_1_Y_Z_^●^ (where Y_Z_^●^ is a tyrosyl radical) [12], for which two isomeric *S*_1_ states can coexist as a consequence of a quasi-reversible structure change induced by proton migration [13]. Two *S*_1_ conformations, closed and open, resembling *S*_2_ states, have been proposed [14]. Due to the orientational Jahn–Teller isomerism, they could convert to each other (open with LS = 1 and closed with HS = 3), and this would determine which Mn(III), Mn1, or Mn4 is oxidized [15,16,17,18]. Two *S*_0_ isomers (Mn(III, IV, III, III) with relatively similar energies analogous to the open and closed states of *S*_2_ were also considered. This time, a singly protonated O5 was assumed in both structures and assigned as a slow H_2_O-exchanging substrate [19,20], but the open form of *S*_0_ was considered preferable [21]. In the subsequent steps of cyclic water oxidation, the *S*_3_ state, activated by two flashes, also shows isomerism. This is probably closely correlated with the two different conformations of the *S*_2_ state. In the *S*_3_ state, all manganese ions remain oxidized Mn(IV), but depending on whether Mn4(IV) is five- or six-coordinated, the Mn_4_CaO_5_ complex adopts different spin and conformational states, respectively, as either an LS state (S = 3), with a stable closed cubane form, or an HS state (S = 6), with a stable open cubane form [17,22,23,24]. Thus, the *S*_1_, *S*_2_, and *S*_3_ states can adopt at least two potentially stable conformations, while it is proposed that the *S*_0_ state exists rather than an open cubane [21,25].

To unravel the mystery of the mechanism of O-O bond formation and oxygen release, it seems crucial to know the last of the metastable states of the Kok cycle, i.e., the *S*_3_ state. Therefore, knowing the individual stages of *S*_2_ → *S*_3_ transition is as important as understanding the possible intermediate *S*_4_ states during the flash that triggers *S*_3_ → (*S*_4_) → *S*_0_ transition [39,40,41,42,43]. It is now known that any electron uptake from Mn_4_CaO_5_ is preceded by the oxidation of TyrZ (Y_z_) and its deprotonation [36,44,45] and that the accumulation of positive charges on the manganese cluster during the transitions between *S_i_* → *S_i_*_+1_ states requires prior proton release [36,46,47,48,49,50,51,52,53]. The only exception is the transition from *S*_0_ → *S*_1_, when the transfer of electrons to Y_Z_^●^ precedes deprotonation [52,53]. There is consensus that during *S*_0_ → *S*_1_ and *S*_1_ → *S*_2_ transitions, oxidation is concentrated on Mn [54,55], but there is no such consensus for *S*_2_ → *S*_3_ transition, where some groups favor an interpretation of the data as indicating Mn-centered oxidation [23,56,57,58] and others suggest ligand-centered oxidation [59,60,61,62,63]. Furthermore, opinions remain divided on proton release during *S*_1_ → *S*_2_ transition. Since no proton release into the bulk has been observed, it is generally assumed that no proton release occurs during this transition [36,43,44,45]. However, during this transition, both theory and experiments suggest that the OEC is deprotonated and H^+^ is taken up in the water clusters, thus preventing the release of protons to the bulk [48,49,52,64,65,66,67].

There are several experimental approaches, combined with computational ones, that attempt to explain how the different conformational forms of the *S_i_* states, in particular *S*_2_ and *S*_3_, can affect the mechanism of oxygen release and its efficiency. At present, two pathways of O-O bond formation involving Mn_4_CaO_5_ are the most considered for reconstructing the Kok cycle. One of them assumes an oxo-oxyl radical coupling mechanism, where the *S*_4_ intermediate state may involve the electrophilic Mn(V)-oxo or Mn(IV)-oxyl radical [46,68,69,70,71]. The other one involves a mechanism of nucleophilic attack by water [29,72,73,74]. A completely different mechanism has been proposed for O-O bond formation within the MnVII dioxo site on Mn4 [75]. Furthermore, different scenarios for the pathway leading to the formation of the O-O bond have been proposed, depending on the assumption of the conformation of the subsequent *S_i_* states and the stage at which the second exchangeable substrate water is bound to the Mn_4_CaO_5_ cluster. For example, one of the models considers additional H_2_O binding during *S*_2_ → *S*_3_ transition and only open structures of *S_i_* states [42], while the other assumes that structural changes in the Mn_4_CaO_5_ complex (open ↔ close) for the *S_i_* states facilitate the coordination of substrate water binding, proton release, and O-O formation [43,75,76,77,78].

The coupling of PSII conformational changes, which can lead to the reorganization of the immediate environment of the OEC, regulating substrate water access, proton release, and ultimately dioxygen formation, and thus affecting its enzyme activity, has been suggested many times; for example, see [78,79,80,81]. In addition to the protein network, the stability/variability of the hydrogen bonds between the water molecules, as well as between the water molecules and the amino acid residues, plays a vital role in this process (for review, see [81]). The presence of more water molecules in the vicinity of the manganese complex, which may be directly or indirectly involved in the oxygen evolution process, was predicted via EPR, mass spectroscopy, or FTIR measurements, among others [50,82,83], and further confirmed in structural studies of the OEC [26,27,84].

**Figure 2:**
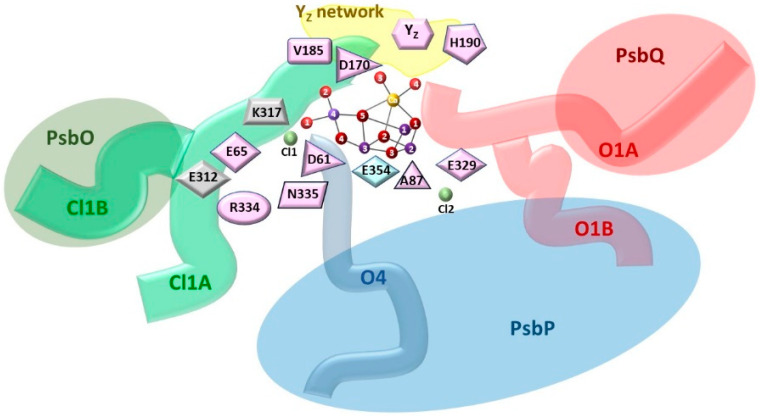
Scheme of water channels identified in a higher plant *Spinacia oleracea* [101]. The location of conserved amino acids [69,89,90,101,112] are indicated (magenta from the D1 core protein; gray—from the D2 core protein and blue—from the inner light-harvesting complex CP43 protein). Areas of the channels partially stabilized by the outer proteins PsbQ, PsbP, and PsbO are highlighted in light red, blue, and green, respectively. The Mn_4_CaO_5_ cluster is also included in the figure. The purple circles represent manganese atoms, the dark red circles oxygen atoms and the light red circles oxygen atoms from bound water. All are numbered according to generally accepted convention. The calcium atom is marked in yellow.

Due to the cyclic nature of the light-driving force-dependent operation of the Mn_4_CaO_5_ cluster and its location and protection by external proteins on the lumenal side of the thylakoids, it has been suggested that the pathways for the entry and exit of substrates (water molecules) and products (protons and O_2_) may be essential for the efficient functioning of PSII [85,86,87,88]. To date, based on theoretical studies, three water channels have been identified that have counterparts in cyanobacteria, algae, and higher plants [89,90]. Two of them, large and broad, are branched and, using the nomenclature based on the entry point of each channel into the OEC region, are also known as the O1 and Cl1 channels, respectively. The third one is a single narrow channel, also known as the O4 channel. In cyanobacteria, the O4 channel is formed by residues D1, D2, CP43, CP47, PsbO (a 33 kDa external protein), and PsbU (an external protein subunit). It connects O4 to the lumen with the participation of protonated D1-D61. But on the other side of D1-D61, there is the Cl1 channel that is at the interface of D2 and PsbO subunits [27,91,92]. The Cl1 channel with its branching arms is indicated as an H-channel rather than a water delivery path [92,93,94,95], although the latter function cannot be ruled out either [27,32]. The O1 water chain, a branched network, is formed in cyanobacteria by the same protein subunits as the Cl1 channel, with one difference: instead of PsbO, the PsbV subunit is involved. This channel is thought to remove O_2_ and/or deliver water to the OEC [91,96,97,98]. On the other hand, the sometimes recognized so-called back channel appears to be inaccessible to water but permeable to O_2_ [99,100]. Hydrophobic channels could be an effective way to remove O_2_ [91]. Among the many water channels reported in cyanobacteria or algae, the organization of amino acids of the Cl1 channel starting at Mn4, W1, W2, and W3 is the most evolutionary conserved. The recognized proton gate residues D1-E65/D2-E312/D1-R334/D1-N335 associated with the proton network rearrangement along this channel were found in all photosynthetic species [89]. The O4 channel is also very conservative at its entrance to the manganese complex near O4 and W1, including D1-D61, but shows a different orientation at its end near the surface. The O1 (large) channel, like the O4 channel, shows a high degree of conservation in the vicinity of the Mn_4_CaO_5_ complex, reaching Ca, O1, and W4, but at the same time, has different orientations at the end of the path close to the bulk, as well as different degrees of branching. For more details, see: [89,90,101]. In plants, however, fewer PSII subunits form the channels mentioned above. The subunits D1, CP43, PsbP (23 kDa external protein), and PsbQ (17 kDa external protein) are involved in forming the O1 water chain, D1, D2, CP43, CP47, and PsbP are involved in forming the O4 water chain, and only three subunits D1, D2 and PsbO (33 kDa external protein) are involved in forming the Cl1 water chain [90,101]. The small radius of the O4 channel (~1.4 Å) in all species suggests that water molecules are also arranged as a single chain in higher plants as well [90]. A rigid water chain with particularly strong hydrogen bonds between the initial water molecules near the Mn_4_CaO_5_ cluster and with its O4 atom indicates that the narrow channel can transport protons via the Grotthuss mechanism since the activation energy of proton transfer is lowest when all water molecules are strongly bound in the H-bond network [53,102,103]. Proton release during the *S*_0_ → *S*_1_ [42,53,103,104] and *S*_2_ → *S*_3_ transition via this channel was proposed [71]. However, the involvement of the O4 channel in the removal of protons during *S*_2_ → *S*_3_ transition was considered to be rather unlikely [42,89], and the Cl1 channel has been proposed as a proton release pathway during this transition [105,106,107,108], with D1-E65 (branch Cl1A) serving as the gate for proton transport to minimize the back reaction [40,89]. Recently, this has been confirmed experimentally through the use of pump–probe time-resolved femtosecond crystallography (TR-SFX) [36]. On the other hand, the O4 channel is suggested to be responsible for supplying water to the OEC during *S*_4_ → *S*_0_ transition [77]. Compared with the binding of ammonia to Mn_4_CaO_5_, water with similar structural and electrical properties was suggested to be supplied to Mn4 also through the O4 channel during *S*_2_ → *S*_3_ transition [100,109,110]. Especially in spinach, it is expected that the O4 channel is able to deliver water efficiently to the manganese complex. This is because the entrance to the channel is wider due to the presence of Ala in D1 at position 87 than in cyanobacteria, where Asn is found [111,112]. The competing hypothesis is that during the transition from *S*_2_ to *S*_3_, a water molecule is introduced into Mn_4_CaO_5_ through the O1 channel on the Ca side [40,42,44,89,109,113], and it could be a precursor of the O6 oxygen occurring in the manganese cluster [36]. This channel has been shown to exhibit the highest water exchange rate [89]. It has also been proposed that in cyanobacteria, the O1 channel may supply water, and both O1 and Cl1 channels release protons in *S*_2_ → *S*_3_ and *S*_3_ → (*S*_4_) → *S*_0_ transitions. But during *S*_0_ → *S*_1_ transition, the O4 channel has been suggested to be the proton exit pathway, which was found to be disconnected in the *S*_2_ state and restored only in the *S*_0_ state [42]. Recently, an interesting observation was made. Namely, glycerol (commonly used as a cryoprotectant and stabilizer of isolated PSII) incorporated into the O4 channel in cyanobacteria at a distance > 10 Å from the OEC affects the LS stabilization of the *S*_2_ state of the Mn_4_CaO_5_ complex, which adopts the ‘open’ conformation (Figure 1b) as a result of disruption to the hydrogen bond network involving D1-D61 when it remains protonated. In the absence of glycerol (D1–61 becomes deprotonated), both states of *S*_2_, i.e., LS and HS (open and closed conformations), are virtually isoenergetic [114]. An analogous effect of regulation of the Mn_4_CaO_5_ complex by allosteric interactions may also occur in higher plants, as may be indicated by the usual occurrence in their case of both spin states of the *S*_2_ state and the disappearance of the HS signal with an increase in the concentration of glycerol [115]. A diagram of the distribution of water channels identified in a higher plant (spinach), with an indication of the location of conserved amino acids, is shown in Figure 2.

The search for water, proton, and O_2_ transport pathways has been and continues to be carried out through the use of in silico experiments with various computational methods, including QM (quantum mechanics)/MM (molecular mechanics), MD (molecular dynamics), and CE (continuum electrostatics)/MC (Monte Carlo studies) using available PSII structures, even the PSII thylakoid membrane model [116]. Various research groups have pointed out similar patterns of water channels in cyanobacteria, but their purpose cannot always be clearly determined. Some counterparts have been found in higher plants, but even minor differences from cyanobacteria may be relevant to their distinct control of protons and O_2_ output during water oxidation or water delivery to the OEC. Identifying these mechanisms is a challenge. Learning about them is the key to achieving a complete picture of how OEC and PSII function as a whole. In general, water diffusion tends to require water-filled channels. Each water channel can potentially evacuate O_2_, but its diffusion can also occur through hydrophobic pathways. It has been suggested that lipid clusters within PSII, due to their predominantly hydrophobic nature, may serve as an oxygen drain and mediate efficient, PSII-safe, and rapid release of O_2_ [94,117]. A highly conserved small hydrophobic pathway in cyanobacteria and algae (prokaryotes and eukaryotes) has been identified at the beginning of the O1 channel and has been suggested to be responsible for facilitating O_2_ release from the OEC [117,118].

As can be seen, the factors regulating the process of water evolution by PSII are extremely complex. To understand them, it is necessary to consider not only the conformation of the OEC and its immediate environment but, most likely, the entire PSII, which is a dynamic system [36,79].

In this study, we focused on investigating the effects of the external proteins PsbO (~33 kDa), PsbP (~23 kDa), and PsbQ (~17 kDa) on the process of oxygen evolution in a higher plant, tobacco. Although the role of these proteins is not yet fully understood, it is known that they play a protective role in stabilizing the binding site of the Mn_4_CaO_5_ complex, ensuring ionic balance in its environment, mainly preventing the loss of calcium and chlorine ions [119,120,121,122,123,124]. In addition, as outlined above, current knowledge suggests that they may be involved in regulating the access of water molecules to the OEC and the removal of O_2_ from it [65,89,90].

In this work, we utilize a fast polarographic measurement technique that enables direct measurement of the kinetics of photosynthetic oxygen evolution without relying on models or assumptions about the initial conditions of the systems under investigation. The primary objectives of this approach are as follows: (i) to examine whether O_2_ evolution is a heterogeneous process, as postulated by previous analyses of the oscillatory pattern of O_2_ release during water oxidation in PSII influenced by short saturating flashes [37,38,125] and (ii) to investigate the impact of external proteins on the kinetics and predicted biphasicity of O_2_ yield in order to gain a better understanding of the factors that govern the efficiency and dynamics of oxygen evolution.

By addressing these questions, the study aims to contribute to the understanding of the complex mechanisms involved in photosynthetic oxygen evolution and the role of external proteins in this process. Understanding the various factors that contribute to the exceptional efficiency of the OEC in water splitting has significant implications beyond the field of photosynthesis research. This knowledge is also important in the design of high-efficiency fuel cells.

## 2. Materials and Methods

The procedures used for the isolation of PSII-enriched thylakoids, PSII BBY, and subsequent removal of extrinsic proteins were carried out according to [126,127] with some modifications [128]. PSII BBY was isolated from freshly harvested laboratory-grown tobacco, *Nicotiana tabacum var. John William’s Broadleaf* (JWB, from Prof. G.H.Schmid’s seed collection) under control conditions (25 °C; humidity, 65%; 14 h day/10 h night; white light—500 µE; red light—20 µE; infrared light; collected leaves from plants 3 months old) in *PSI FytoScope* growth chamber (PSI Photon System Instruments, Drasov, Czech Republic). The culture was carried out in summer. In the original procedure, two consecutive Triton washes were used to obtain a sample with high PSII enrichment. We wanted to maintain a sufficiently large natural pool of plastoquinone in the sample to avoid the need to add external acceptors. For this reason, the second Triton wash series was not carried out. A Triton X-100-to-chlorophyll (Chl) ratio 20:1 was used. The sample was washed four times with a large volume of Hepes II buffer to remove any residual Triton, which could lead to further degradation of the sample during storage. The washing procedure was continued until the supernatant was completely transparent. The PSII membrane (2 mg Chl/mL) was suspended in Hepes II buffer (15 mM NaCl, 5 mM MgCl_2_, 20 mM Hepes, and 400 mM sucrose; pH 6.5). The two extrinsic proteins, PsbQ (~17 kDa) and PsbP (~23 kDa) were removed from PS II BBY via incubation in a buffer containing 1.5 M NaCl, 400 mM sucrose, 40 mM Hepes, and 5 mM MgCl_2_ (pH 6.5) for 30 min at 0 °C (kept on ice). To remove three extrinsic proteins, PsbQ, PsbP, and PsbO (~33 kDa) instead of NaCl 1.5 M MgCl_2_ was used. The samples contained 1 mg Chl/mL. After treatment with the high concentrations of sodium and magnesium salts and two washings in Hepes II, the PSII membranes were resuspended in the same medium. Our experience [128], as well as that of other groups [for example, [127]], shows that 1.5 M MgCl_2_ and CaCl_2_ are similarly efficient in removing the three extrinsic proteins from PSII, but CaCl_2_ induces a much greater inhibition of oxygen evolution than MgCl_2_. Although both the CaCl_2_ and MgCl_2_ washes do not remove Mn from the OEC, the oxygen evolution in the samples treated with high concentrations of CaCl_2_ shows more than twofold greater decrease in the activity of PSII in the oxygen evolution than in the case of MgCl_2_. As shown by the data presented in [127,128], the use of CaCl_2_ would make it virtually impossible to perform a similar analysis of oxygen evolution. Since the samples showed the expected activity, the active site had to contain bound Mn ions. There was no need to add additional Ca^2+^ ions to the buffer as we were not using chelators. Furthermore, these would induce additional OEC modifications, which we wanted to avoid.

All of the prepared samples were divided into small portions and frozen in liquid nitrogen and stored at −80 °C until measurement (no longer than three months).

Samples lacking the two outer proteins PsbP and PsbQ and the three outer proteins PsbO, PsbP, and PsbQ are referred to in the paper as PSII BBY—P,Q and PSII BBY—O,P,Q, respectively.

To confirm protein removal, denaturing SDS-PAGE was conducted using a vertical polyacrylamide gel system in a Mini-Protean Tetra Cell apparatus (BioRad, Hercules, CA, USA) according to the standard protocol [127], as shown in Figure S6 in the Supplementary Materials.

### Fast Polarography Experiments

Amperometric measurements of oxygen evolution in PSII BBY untreated and PSII BBY—P,Q and PSII BBY—O,P,Q were performed under short saturating flashes. We did not use any external acceptors because our experience and other groups (see, e.g., [39]) show that different external acceptors modify the O_2_ yield pattern in different ways. We also did not activate the sample with a single flash. This also affects the distribution of the *S_i_* states and other initial conditions of the samples. The assumption that only the *S*_1_ state is occupied is not necessarily true. Its stabilization also depends on other factors affecting the donor and acceptor sides of PSII in the dark (see Section 4). Furthermore, one had to assume that the transition from *S*_0_ to *S*_1_ must occur with probability 1, i.e., there are no misses, i.e., *α*_0_ = 0. Secondly, the sample exposed to a saturating flash of light changes the system, and even assuming the validity of the assumptions made, this certainly cannot be applied to the BBY PSII—O,P,Q sample, which means that the results obtained could not be compared for all samples.

A three-electrode system (a Joliot-type electrode, descrbed in [129,130]) was used. The polarization voltage was set at −680 mV. A xenon lamp (X-strobe. Perkin Elmer, Salem, MA, USA) emitted flashes of ~3 μs half-width. According to the manufacturer, the spectral bandwidth of the flash lamp is 250–1100+ nm, with infrared radiation accounting for 16% of the light output for wavelengths between 800 nm and 1100 nm in the total spectrum of the xenon lamp. The lamp is equipped with an optical fibre with a 13 mm diameter. It is placed at a distance not exceeding 1 cm from the electrode surface. The approximate light output from the fibre optic light guide is about 150 mJ per flash per cm^2^ (~2 J input energy per flash). With a realistic assumption of 70% luminous flux at the surface of our electrode and an average conversion factor for daylight, one may estimate that this gives about 500 µmole/cm^2^ per flash (about 98% accumulated in the peak of the flash). We verified that changes in the distance of the optical fibre from the surface within 1 cm while maintaining the other measurement parameters had no effect on the observed oscillations of the O_2_ yield under these short flashes. The average O_2_ evolution pattern, determined from three independent measurements, was maintained for each of these distances. This means that there was no change in the distribution of the initial *S_i_* states and the miss parameters. Thus, under our experimental conditions, we may assume that the light is saturating. The surface of our electrode on which the sample is spread is about 1.13 cm^2^ (its external diameter is about 1.2 cm). The sample is evenly distributed on the electrode surface, forming a very thin layer after sedimentation on a special Millipore filter of the same size as the electrode. Thus, under these experimental conditions, we may assume that the light is saturating. The volume of buffer (Hepes I) above the sample was about 600 µL.

**Figure 3:**
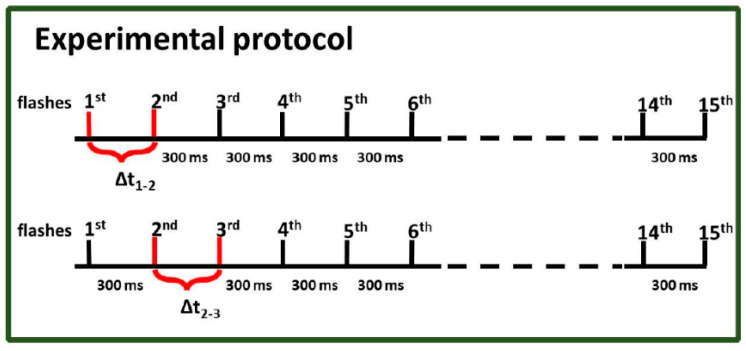
The experimental protocol for oxygen evolution experiments. The time separation between the 1st and the 2nd (Δ*t*_1–2_) or the 2nd and the 3rd flash (Δ*t*_2–3_) varied from 5 m to 500 m.

**Figure 4:**
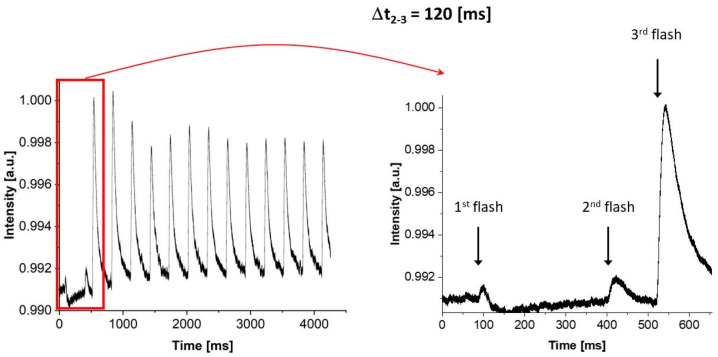
Example raw data for PSII BBY_control_ for Δ*t*_2–3_ = 120 m. The experimental conditions were as described in the text.

The experimental protocol shown in Figure 3 was designed to directly observe the heterogeneity of oxygen evolution in PSII. Two independent experiments were performed in which (i) the intervals between flashes 1 and 2 were varied (as shown in Figure 3—upper scheme), and the remaining 14 flashes were delivered at 300 m intervals, or (ii) the intervals between flashes 2 and 3 were varied (as shown in Figure 3—lower scheme), and the remaining 14 flashes were delivered at 300 m intervals. To illustrate the course of the experiment, Figure 4 shows example raw data for PSII BBY_control_ for Δ*t*_2–3_ = 120 m. It is the simplest and most natural approach to monitor kinetics of oxygen evolution under the third flash (*Y*_3_) as a function of Δ*t*_1–2_ or Δ*t*_2–3_. The idea was to check if the observed kinetics for these two protocols were different or the same. The first scenario would imply a bifurcation of the oxygen evolution process between these transitions, while the second one that linearity is maintained between the transitions, and that the classic Kok’s model can be applied.

Each measurement for a given fixed period Δ*t*_1–2_ or Δ*t*_2–3_ (varied from 5 m to 500 m) was performed on a freshly applied sample, previously incubated in the dark, as described above. In a control protocol typically used as a standard, all flashes were 300 m apart. The final volume of samples containing about 30 µg Chl suspended in a Hepes I buffer (15 mM NaCl, 5 mM MgCl_2_, 20 mM Hepes, pH 6.5) was 500 μL. Thus, the concentration of chlorophyll in the polarographically measured samples was 60 µg/mL. After 7 min of dark incubation on ice (2 min) and sedimentation on the filter at room temperature (5 min), the samples were transferred to the electrode in dim light conditions and were incubated for another 10 min in the dark. This time was sufficient for the electrode signal to stabilize at a constant level. A thawed portion of the sample was used within approximately 1.5 h. Once the sample was thawed, it was stored on ice in the dark. The activity and stability of the sample were monitored for a standard period of 300 m between all flashes at the beginning, in the middle, and at the end of the measurement series. For a given series of measurements, variable periods of Δ*t*_1–2_ or Δ*t*_2–3_ were randomly selected. The amplitude of oxygen evolution *Y*_3_ was normalized to the sum of all amplitudes (*Ys*) in each measurement, and then these values were normalized to the mean value of the ratio *Y*_3_(300 m)/*Y_s_* (300 m) obtained for the standard protocol (i.e., Δ*t*_1–2_ or Δ*t*_2–3_ of 300 m) in a given series of measurements. Finally, all of the normalized results from a given series of measurements were scaled to the average of all individual measurements for a given Δ*t*_1–2_ or Δ*t*_2–3_ (normalized as described above) for a range of variable time periods from about 200 m to 500 m in order to show all of the data in one figure, as shown in the Figure in Section 3.2.

Thus, the approach to obtain information on the kinetics of oxygen evolution based on the variability of the periods between the initial flashes is different from the protocols used to measure the lifetimes of the *S_i_* states and the method of their determination, which depends on the model adopted [131,132]. The variable period in these experiments usually ranges from 0.5 s to a few hundred seconds and concerns the dark adaptation between a short series of pre-flashes and the actual sequence of a dozen flashes.

The proposed method of measuring oxygen evolution time using fast polarography has significant advantages over the shape analysis of the electrode response signal. Analysis of the polarographic signal is complex and does not provide direct information on the biphasic nature of O_2_ evolution in PSII. Furthermore, as shown in [133], the signal recorded on the electrode is strongly dependent on the contact of the sample with the electrode or the thickness of the sample, apart from the voltage applied to the electrode. However, the porosity of the sample and/or its external potential, which may change due to sample modification (e.g., protein removal), will also affect the shape of the signal.

Typical shapes of the polarographic signals obtained for the PSII BBY control, PSII BBY—P,Q and PSII BBY—O,P,Q under the same experimental conditions are shown in Figure S5 in the Supplementary Materials.

## 3. Results

### 3.1. Analyzing Oxygen Evolution Patterns

**Figure 5:**
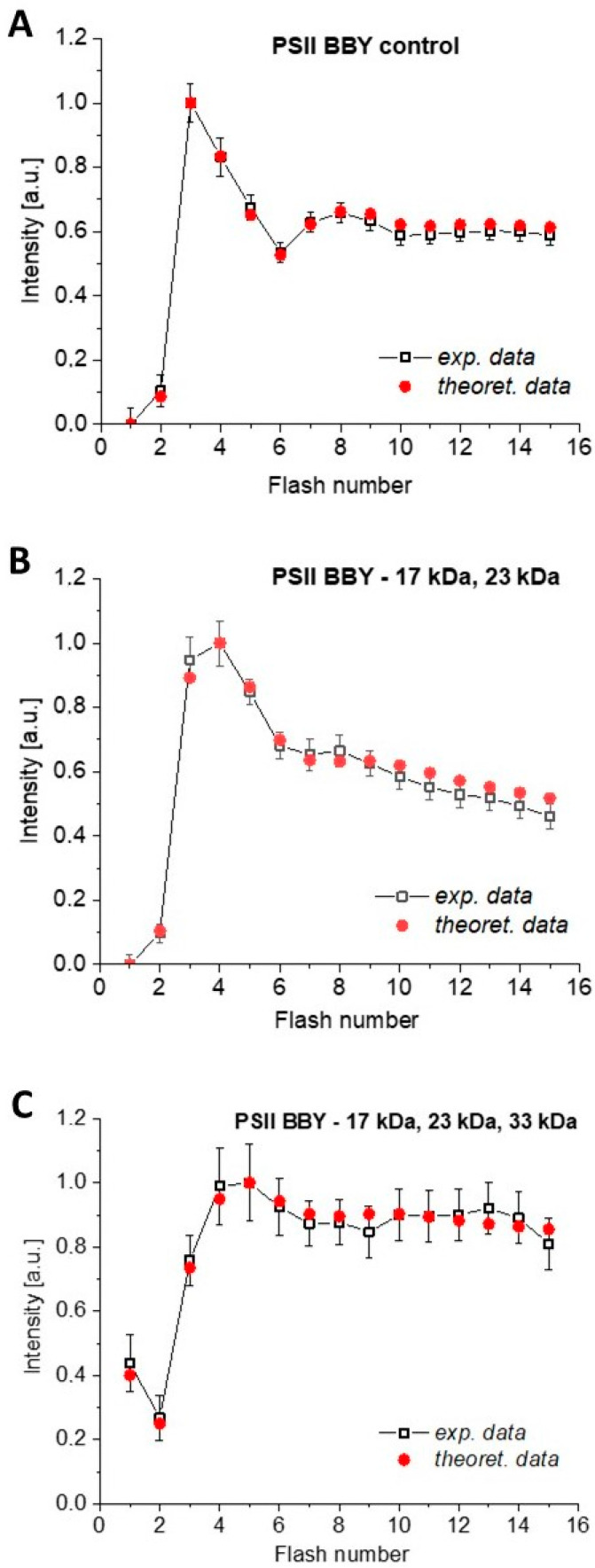
Flash-induced oxygen yield pattern in intact PSII BBY (**A**) and PSII BBY depleted of the extrinsic proteins via NaCl (**B**) and MgCl_2_ (**C**) washing. Samples were suspended in the Hepes I buffer pH 6.5. The flashes were 300 m apart. Amplitudes are always normalized to the amplitude of O_2_-evolution under the third flash. The experimental data are the mean values obtained from 3 independent measurements in each case. The error bars correspond to the maximum error.

In the standard procedure for the measurement of oxygen release under short, saturating flashes using a fast three-electrode system, the time interval between the flashes is 300 ms, which provides optimal experimental conditions. Experimental and theoretical data of the flash-induced oxygen evolution in native and two or three extrinsic-protein-depleted PSII BBY are shown in Figure 5 (example raw data are presented in Figure S1 in the Supplementary Materials). No external acceptors were used in this study to avoid altering the functioning of the active PSII complexes [39]. Therefore, the total oscillatory oxygen release signal slightly decreases with an increasing number of flashes, as one may expect [37,39,132]. This is due to the partial depletion of a natural acceptor (plastoquinone PQ-9), which reduces the number of active photosystems. Typically, the mobile PQ pool is estimated to be 5–10 molecules per PSII RC in thylakoids and does not change significantly in isolated membranes due to the lipophilicity of quinone but is not uniformly distributed [134,135,136]. In our case, this is determined by the *C* parameter, which is 1 for a fully functional system with a sufficient abundance of the acceptor, which means that the number of active centers is maintained (i.e., $\;\sum_{i=0}^{4}S_{i}=1$, where *S_i_* is a fraction of the OEC in a specific oxidized state *i*, Markov chain). We assumed that the decay of the signal follows a geometric progression with the ratio *C*, which is slightly less than one (see Equation (S4) in Supplementary Materials). The quenching of the total signal is stronger in PSII BBY—P,Q than in the control sample (Figure 5) because the removal of two external proteins (PsbP and PsbQ) reduced the available plastoquinone pool. In this case, the *C* parameter has the lowest value (Table 1). However, the activity of PSII BBY—P,Q was only reduced by about 10% compared to intact PSII BBY (Figures S2 and S5a in the Supplementary Materials). In contrast, after the removal of three external proteins (PsbO, PsbP, and PsbQ), when the number of active centers is significantly reduced by about 60% (see Figures S2 and S5a in the Supplementary Materials), the effect of signal quenching due to PQ deficiency is much less pronounced (Table 1 and Figure 5).

The failure rate *α* of the trapping centers (called misses) leads to a redistribution of the *S_i_* states and, consequently, to a damping of the oscillations of the O_2_ release. The original Kok model assumed equal misses for light-driven transitions *S_i_* → *S_i_*_+1_ and additionally doubled effective excitation in a fraction *γ* of the centers, which are in the *S*_0_ and *S*_1_ states (called double hits and also equal). However, it has been shown that when this homogeneous model is used, there are significant discrepancies between the theoretical and experimental O_2_ yield patterns [137,138]. Double hits were introduced to explain the often-significant oxygen release that occurs at the second flash. In addition, double hits lead to an increase in oscillation damping at higher flash numbers. This further reduces the observed differences between experimental and theoretical data but still does not allow for the satisfactory reproduction of the observed O_2_ evolution oscillations. In the Supplementary Materials, Figure S3 shows fits using the Kok model with equal misses or equal misses and double hits to our experimental data of the samples studied here, i.e., PSII BBY control, PSII BBY—P,Q, and PSII BBY—O,P,Q. The fitted parameters are given in Table S1. Various approaches to the modification of the Kok model for a better representation of the experimental data are mentioned in the Supplementary Materials. However, it has been demonstrated that the progressive damping of the oscillations is mainly due to misses, and the heterogeneous model with different *α_i_* misses, omitting double hits, gives a better quantitative agreement with the experimental data obtained for other systems [37,139,140,141,142,143]. Unequal misses for *S_i_* → *S_i_*_+1_ transitions for *i =* 0, 1, 2, 3, 4 were also experimentally proven via EPR measurements [144]. Furthermore, it was found that extending the Kok model to a 5S-state model by explicitly including the *S*_4_ state and introducing biphasicity in the O_2_ evolution by introducing a bifurcation of the *S*_3_ → (*S*_4_*/S*_4_*’)* → *S*_0_ transition (Figure 1c) resulted in an excellent reproduction of the experimentally observed patterns of O_2_ release [37].

The distinction between the short-lived and the metastable *S*_4_ state allowed us to estimate the contribution of PSII involved in the fast (*d*) and slow (1 − *d*) phases of oxygen evolution; for example, see the scheme in Figure 1c and Section 4. Due to its simplicity, the proposed model allows all parameters *α_i_*, parameter *d*, and the initial distribution of *S_i_* states, to be fitted to the experimental data without any assumptions. This results in higher accuracy and precision than the classical Kok models (see Figure S3 and Table S1 in the Supplementary Materials). Note that *S*_4_ = 0, and *S*_3_ = 1 − *S*_0_ − *S*_1_ − *S*_2_ leaves eight free parameters to be fitted. However, the initial conditions are sufficiently well defined. The first four amplitudes are a good approximation of the initial *S_i_* states. Consequently, in our model, the minimum of the fit quality as a function of the parameters used is stable. The sensitivity of the heterogeneous model to *α*_i_ parameters is presented and discussed in the Supplementary Materials (Figure S4).

The results of the theoretical evaluation of the experimental data for the control sample, PSII BBY—P,Q and PSII BBY—O,P,Q obtained using the 5S-state heterogeneous model are shown in Table 1. It is worth noting that the same trends in changes in initial *S_i_* state occupancy and misses are also obtained for the control sample and samples lacking two or three external proteins using the Kok model with equal misses and equal misses and double hits (see the Supplementary Materials, Table S1, Figure S3). As can be seen, these homogenous models do not allow for the satisfactory reconstruction of the oxygen evolution oscillations, especially in the case of the control sample and PSII BBY—P,Q. The quality of the fit compared to the extended 5S-state model is about 6–13 times worse in the first case and about 2–5 times worse in the second case. Due to the low signal intensity and increased oscillation damping observed for PSII BBY—O,P,Q, the fit quality parameter is only about 1.5 times worse than with the extended 5S-state model. The use of the heterogeneous 5S state model allows for a more detailed analysis of the causes of the decrease in the probability of transitions between the *S_i_* → *S_i_*_+1_ states.

### 3.2. Kinetics of the Fast and Slow O_2_ Release Pathways

To verify the heterogeneity (biphasicity) of the oxygen release process in direct measurement, we used a protocol for measuring oxygen release using a fast electrode type, as presented in this paper (Figure 3 and Figure 4). Two protocol variations were used, one with different periods between the first and second flashes (Δ*t*_1–2_) and the other with varying periods between the second and third flashes (Δ*t*_2–3_).

**Figure 6:**
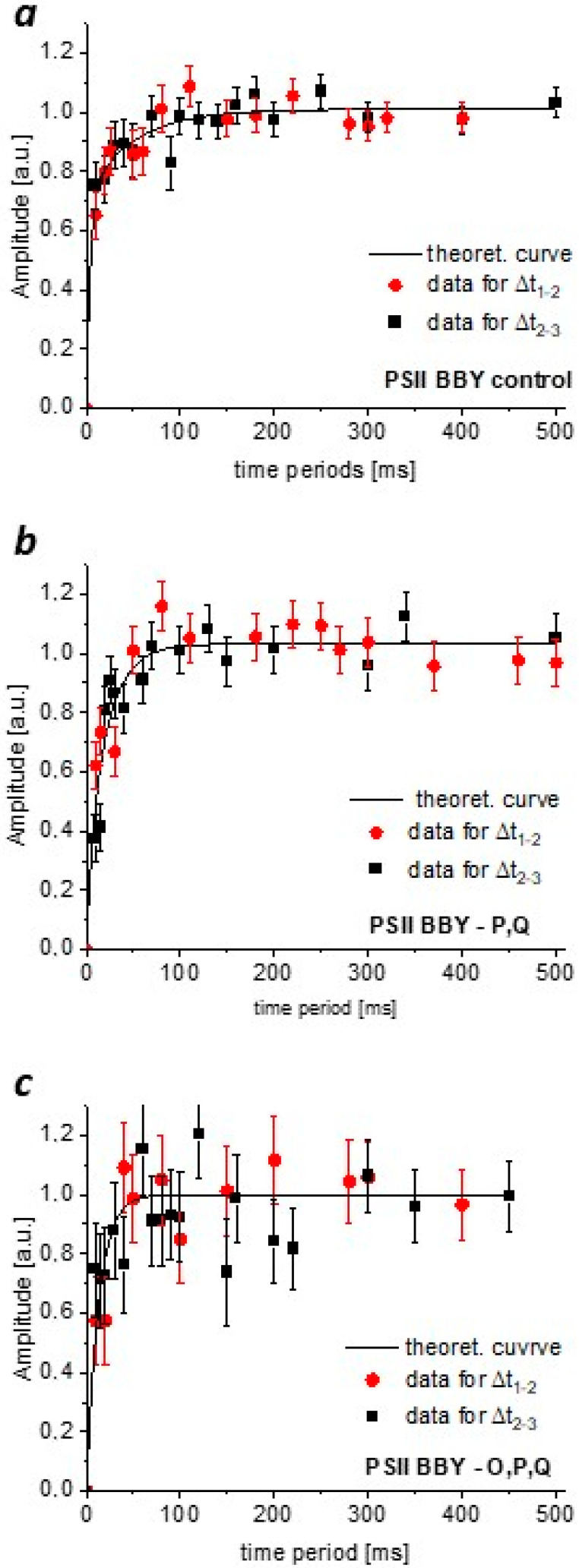
The dependence of O_2_ release under the third flash on the time interval between the first and second flashes, Δ*t*_1–2_, (red symbols) and the second and third flashes, Δ*t*_2–3_ (black symbols) for control PSII BBY (**a**), PSII BBY—P,Q (**b**), and PSII BBY—O,P,Q (**c**). Each experimental point is a mean of at least 3 independent measurements. Error bars represent the root mean square error. The theoretical curve shows the fit obtained using a biexponential function (Equation (1)).

Figure 6 shows the dependence of the changes in the oxygen evolution after the third flash as a function of the flash interval when Δ*t*_1–2_ (red symbols) and when Δ*t*_2–3_ (black symbols) changed. Note that for a given sample, whether the intervals between the first and second flashes or between the second and third flashes were altered, the dependence of the changes in oxygen evolution after the third flash on the flash interval is the same. This is in accordance with Kok’s linear sequence of transitions between the *S_i_* states.

The experimental data were fitted using a dual relaxation function, defined as the sum of two exponential relaxations (solid lines in Figure 6a–c):

(1) $$Y_{3}=A_{fast}\left(1-\exp\left(-\frac{t}{\tau_{fast}}\right)\right)+A_{slow}(1-\exp\left(-\frac{t}{\tau_{slow}}\right),\;$$

where *A_fast_* (*A_slow_*) and *τ_fast_* (*τ_slow_*) are the amplitudes and time constants characterizing the fast (slow) O_2_ release, respectively. For the normalized data, *A_fast_* and *A_slow_* denote the contributions of the individual phases. The parameters *A_fast_*, *A_slow_*, *τ_fast,_* and *τ_slow_* obtained from the data for control PSII BBY and PSII depleted of two or three external proteins are listed in Table 2.

In the case of the control sample and PSII BBY—P,Q, two components were necessary to obtain a satisfactory fit to the experimental data, whereas in the case of PSII BBY—O,P,Q, one component was sufficient. We checked that the behavior of the experimental data obtained for samples depleted of extrinsic proteins could not be satisfactorily reproduced by assuming two phases with time constants as for the control sample (Figure S7 and Discussion in the Supplementary Materials). The contribution of the fast component shows excellent agreement with the values and direction of change in the parameter *d*, corresponding to the fast phase of O_2_ release, obtained from the analysis of the O_2_ yield pattern using the 5S-state model.

Applying this experimental approach, it was also possible to determine the time constants of the fast and slow pathways of oxygen release in the samples. For the control sample, the time constant of oxygen release in the slow (~44 m) and fast (~4 m) phases differs by an order of magnitude. In the case of PSII BBY—P,Q, the difference is only about 3.5 times. This is because the fast phase is slowed down by about 2 m, while the slow phase is twice as fast. The removal of all three proteins resulted in a further acceleration of the slow phase by a factor of about 1.7 (Table 2).

## 4. Discussion

### 4.1. Analyzing Oxygen Evolution Patterns

In the control sample, we observed the highest initial occupancy of the *S*_1_ state (~87%) and comparable occupancies of the *S*_0_ and *S*_2_ states at about 5% and 8%, respectively. The deletion of the PsbQ and PsbP proteins did not affect the initial distribution of the *S_i_* states. It is known that during prolonged darkness, the OEC is mainly in the *S*_1_ state [145]. This is due to the oxidation of the *S*_0_ state by an electron carrier, TyrD^+^ (tyrosine Tyr160 of peptide D2, Y_D_ in Figure 1) [7,32,61,93,146,147,148]. Moreover, it has been proposed that *S*_1_ is stable in the dark because the oxidation of Mn3(III) to Mn3(IV) forces the deprotonation of a μ-hydroxo group at the O4 position in the Mn_4_CaO_5_ cluster, and the proton is transferred along the O4 water channel up to ~13.5 Å from O4 [53]. So, the *S*_1_ state does not return to the ground state of *S*_0_ in the usual experimental time of a few minutes. Consequently, the occupancy of the *S*_1_ state in darkness is dominant, and therefore, the first maximum is observed under the third flash. A low occupancy of the *S*_2_ state, i.e., a low O_2_ yield under the second flash, is observed in a number of samples. Even shortening the duration of the flash at mid-maximum intensity from µs to ns did not result in the disappearance of oxygen release under the influence of the second flash [149,150]. For a long time, it was thought that the *S*_2_ state could not be stable after long dark incubation and that only the *S*_0_ and *S*_1_ states would be stable in the dark. Moreover, reduced TyrD, accumulated in the dark due to its involvement in the oxidation of the *S*_0_-state, *S*_0_TyrD_oxd_ → *S*_1_TyrD_red_, has also been shown to be able to reduce *S*_2_ and *S*_3_ states [151,152]. However, the efficiency of electron transfer from the reduced TyrD to the *S*_2_/*S*_3_ states depends on the protonation in the vicinity of the TyrD and the OEC. It was observed that the oxidized TyrD present in the *S*_1_ centers showed high stability, although its slow reduction was detected in the dark, most likely via *S*_1_ TyrD_oxd_ → *S*_2_ TyrD_red_ transition [147]. This would explain why, in the absence of electron donation from the acceptor side, a small population of the *S*_2_ state is always present in samples with long dark adaptation. This is our case. The higher states are expected to be unstable [37,153,154].

The additional removal of the PsbO protein significantly affected the initial distribution of the *S_i_* states. On the one hand, this decreased the stability of the *S*_1_ state in the dark and, on the other hand, increased the stability of the *S*_2_ and *S*_3_ states. This observation is consistent with the fact that PsbO is an already-known MSP protein (i.e., manganese complex stabilizing protein). Most probably, it is responsible for stabilizing not only the Mn_4_CaO_5_ complex but also the surroundings of the cluster, including TyrZ and TyrD and the entire hydrogen network in their vicinity [155]. The effect of increased stability of the higher states caused by the PSII depletion of PsbO has also been observed by other groups [156,157] but has not been discussed. We suspect that these changes are related to increased uncontrolled water molecule access to the OEC and modification of the surrounding hydrogen network.

In PSII BBY—P,Q, a significant decrease in transition efficiency between the *S_i_* states was observed (Table 1 and Table S1). There was an increase in the parameter *α_t_* (total miss: $\alpha_{t}=\sum_{i=0}^{3}\alpha_{i}$) mainly due to an increase in *α*_0_ and *α*_1_, while the parameter *α*_2_ remained unchanged. The value of parameter *α*_3_ also increased, indicating that about 88% of the *S*_3_ states efficiently transitioned to *S*_4_ states. The probability of this transition in the control sample was nearly 100% under our experimental conditions. At the same time, the contribution of the fast mode to the O_2_ yield decreased (parameter *d* decreased almost threefold compared to the control sample). The removal of all three external proteins did not affect the transition probability between *S_i_* → *S_i_*_+1_ states compared to PSII BBY—P,Q. However, the removal of the additional PsbO protein affected the initial distribution of the *S_i_* states and further reduced the *d* parameter to a value of about 0.02, indicating almost complete inactivation of the fast O_2_ release channel.

It is noteworthy that in the case of the control sample, the misses came almost exclusively from *α*_2_, which maintained its value in samples lacking outer proteins of two or three, giving the highest contribution to *α_t_*. The lowest efficiency of the transition *S*_2_→ *S*_3_ is consistent with the unique character of this transition, confirmed via various measurements sensitive to the reorganization and charge transfer changes during this step of the cyclic transformation of the OEC [44,71,78,89]. EXAFS experiments implied significant structural changes during *S*_2_ to *S*_3_ transition, observing the Mn-Mn and Mn-Ca distances [48,158]. The significant value of the *α*_2_ miss parameter may also reflect the structural changes observed around TyrD under two-flash illumination. This is due to the partial oxidation of TyrD resulting from inefficient electron donation from the Mn_4_CaO_5_ complex, as suggested in [71]. In addition, FTIR measurements showed that the binding of one of the substrate water molecules to the Mn_4_CaO_5_ complex occurs during *S*_2_ → *S*_3_ transition and the other during the *S*_3_ → (*S*_4_)→ *S*_0_ transition [36,50,159]. In addition, it has been suggested that releasing a proton during *S*_2_ → *S*_3_ transition could be a rate-limiting step in this transition [108]. Mn_4_CaO_5_ binds four water molecules. Two water molecules, called W1 and W2, are ligated to Mn4, and two others, W3 and W4, to Ca (Figure 1b). During *S*_2_ to *S*_3_ transition, additional bridging oxygen (O6) ligated to Mn1 near O5 was observed [42,44,71,78]. The sixth oxygen bound to Mn1, presumably as an OH group, may originate from substrate water directly bound to Mn1 earlier in *S*_1_ → *S*_2_ transition [48] or from a water molecule already present in the *S*_1_ state, proposed to be a Ca (W3) [113,160,161] or Mn4 (W1) ligand [110,162,163]. In the latter case, when an additional water molecule is bound to the Ca or Mn4 ion (which requires Mn4(IV) in the *S*_2_ state) during *S*_2_ → *S*_3_ transition, one of the internal water molecules (W1 or W3) moves to a new position, i.e., O6 [76,164,165]. However, recently, a completely new scenario has been proposed based on TR-SFX experiments, which can be used to follow PSII structural dynamics in the ns to ms timescale [36]. The O6 ligand may have its origin in a water molecule from the outer sphere of the water lattice. It binds to the calcium ion in less than a microsecond during *S*_2_ → *S*_3_ transition. However, this is preceded by a significant reorganization of the Mn_4_CaO_5_ cluster and the surrounding proton network, including water molecules.

It is currently not possible to determine whether the heterogeneity in the structures of the *S_i_* states, described in the Introduction, indicates multiple pathways for O-O bond formation, or rather a single pathway within a given reaction center that is optimized during the cycle, leading to O_2_ release [25,43,48,76,77,80,166,167]. In the latter case, the different conformations of the *S_i_* would be transition states that occur in a single chain of events in the Kok cycle. For example, the topology of a high-spin closed cubane in the *S*_2_ state (formed, e.g., when H^+^ is released from the *S*_2_Y^●^ state) was proposed to be essential for the transition to the HS *S*_3_ (closed cubane) state, which changes to the low spin LS *S*_3_ (open cubane) state on the addition of H_2_O [35,168]. It is often suggested that the emergence of a high-spin closed cubane topology in the *S*_2_ state is of mechanistic importance for the subsequent catalytic steps [45,163]. However, the specific steps leading to the formation of O-O and the release of O_2_ may be much more complicated or different from those mentioned above. For example, the observed HS *S*_2_ may not result from conformational changes in the Mn_4_CaO_5_ complex but from the protonation of O4 in the open cubane form [169]. Proton isomerism of the *S*_2_ state has been independently suggested in [161]. Proton isomerism of high- and low-spin *S*_3_ states has also been proposed [80,170].

Furthermore, the question arises as to the causal relationship between the changes in the manganese complex itself and the protein network of its proximal and distal environment. This point is crucial because of the need to synchronize the delivery of water, the reception of protons, and the release of O_2_ with the cycling of the Mn_4_CaO_5_ complex. Obtaining a complete picture of the process of OEC functioning is hampered because of, among other things, the insufficient resolution of PSII structures, the blurriness of the images obtained from them, the difficulty of capturing the various stages of the Kok cycle (mixing of *S_i_* states), the influence of the measurement conditions on the stability of the sample and the oxidation states of the manganese cluster or the preparation method on the functioning of PSII, the degree of its hydration, and simplified theoretical models applied [36,77,171,172,173,174,175,176,177,178].

Based on the existing knowledge, one may suggest the possible causes of the changes in the *α_i_* parameters due to the extraction of the two outer proteins, PsbP and PsbQ. As a first approximation, the removal of these two external proteins should primarily be attributed to proton channel dysfunction. This assumes that the *S*_0_ → *S*_1_*, S*_1_ → *S*_2_*, S*_2_ → *S*_3_ and *S*_3_ → (*S*_4_) → *S*_0_ transitions primarily require efficient proton extraction to minimize ‘back reactions’, mainly related to electron flow. This will, of course, be accompanied by changes in the coupling efficiency within the Mn_4_CaO_5_ complex, as well as changes in its interaction with the immediate protein–water environment. They will certainly be reflected in the conformation and stability of the subsequent *S_i_* states resulting from these transitions. Referring to the proposed contribution of PsbP in stabilizing O1B and O4 channel outlets and PsbQ in stabilizing the O1A channel outlet, recognizing in higher plants [90,101] (see Figure 2), the observed increase in miss parameters very well reflects the changes in the activity of these channels. Thus, the lack of regulation of proton uptake at the output of the O4 water channel, which has been indicated to be the main, if not the only, proton transfer channel for the *S*_0_ → *S*_1_ transition [42,53,61,104], translates into a high increase in *α*_0_. The dysfunction of both the O4 channel and the two branches of the O1 channel leads to an increase in *α*_1_ and *α*_3_ but to a much lesser extent. For *S*_1_ → *S*_2_ and *S*_3_ → *S*_0_ transitions, *α*_1_ and *α*_3_ are approximately 2 and more than 4 times lower than *α*_0_, respectively. This may indicate a smaller contribution of the O4 channel to deprotonation processes during these transitions, especially in terms of *S*_3_ → *S*_0_ transition, and a larger contribution of the other channels. It has been suggested that the O4 channel only opens during *S*_0_ → *S*_1_ transition [42]. It is also likely that the smaller changes in O1 channel function induced by the deletion of the PsbQ and PsbP proteins are due to the specificity of this channel, which is wider than the others. It contains highly mobile water molecules along almost its entire length and may function primarily as a water supply channel [89]. This has recently been experimentally demonstrated for water binding during *S*_2_ → *S*_3_ transition [36]. Since the extraction of all three outer proteins, i.e., PsbQ, PsbP, and PsbO, did not further alter the parameters *α*_0_, *α*_1,_ and *α*_3_, it can be expected that mainly the Cl1A branch (Figure 2) is responsible for the reception of H^+^ during *S*_1_ → *S*_2_ and *S*_3_ → *S*_0_ transitions. It is also the most evolutionarily conserved water channel, also in higher plants [179,180,181,182]. It contains the very important conserved amino acid sequences E65/E312/R334, which are suggested to be gates that regulate the release of protons into the lumen [183,184] as well as being possibly involved in the uptake of water (see Figure 2) [100,185]. Because no proton release to the bulk is observed during *S*_1_ → *S*_2_ transition [51,186], it is proposed that, in this case, the proton is temporarily stored in the form of hydronium in the hydrogen bond network in the vicinity of Ca [64,172] or in the Cl1 channel to which it can be transferred via D61 [182]. For example, Asp 170 has been suggested to be involved [69] in this process, but other deprotonation pathways of Mn_4_CaO_5_ cannot be excluded. Indeed, the formation of a cationic water cluster was observed, which indicates transient storage of the proton in the *S*_2_ state in the form of the nH_2_O(H_3_O)^+^ cluster, where n = 5 [65]. It should be noted here that the proton network of the Cl1 channel, which connects to the O4 channel in the vicinity of O4 of the manganese cluster, extends through a network of hydrogen bonds involving unbound and bound water molecules on the Mn_4_CaO_5_ cluster and neighboring amino acids up to TyrZ [27,42,91]. On the opposite side of the manganese complex, close to Ca, water molecules can penetrate from the O1 channel [42] (Figure 2).

The highest value of the miss parameter assigned to *α*_2_, regardless of the presence of external proteins, is related to the specific nature of *S*_2_ → *S*_3_ transition. As mentioned above and shown in recent experiments [36,40,41], this transition requires large rearrangements of the Mn_4_CaO_5_ cluster associated with substrate water binding. Water availability and binding it to a specific site for the required conformation of the complex may be the main reason for the low efficiency of this transition [185]. In particular, the transport of a water molecule from the water network of the O1 channel to the Mn_4_CaO_5_ cluster, which requires a coordinated reorganization of the hydrogen network beyond the first or even the second coordination sphere of the manganese complex and results in the attachment of an additional water molecule to the calcium ion, as proposed in [36], could be a critical step in the oxygen evolution process. Thus, mere deprotonation of Ca-bound H_2_O involving the Cl1 channel, as proposed in [36], would not be the least efficient step for the *S*_2_ → *S*_3_ transition [43,44,109,187,188,189]. The independence of *S*_2_ → *S*_3_ transition from the presence of PsbQ, PsbP, and PsbO may also suggest that Cl1A may act as a water transport channel during this transition. This is consistent with previous predictions [184]. On the other hand, if access to substrate water is not a bottleneck for this transition, then perhaps the assumption of a mechanism (‘carousel’ or ‘pivot’) implying that the O6 position is occupied by one of the internal waters already present in the manganese cluster is still possible. Then, the high stabilization of the *S*_2_ transient state before the electron flow to TyrZ and/or the instability of the *S*_3_ state would explain the low efficiency of this step of the Kok cycle. The much higher probability of the *S*_3_ → (*S*_4_) → *S*_0_ transition than in the previous step suggests that, in this case, the problem is not water access but deprotonation and probably O-O bond formation, the initial stage of which begins with the formation of the *S*_3_ state [36,40,41], i.e., during *S*_2_ → *S*_3_. This could mean that during the formation of the *S*_0_ state, the water molecule is already attached in the immediate vicinity of the Mn_4_CaO_5_ cluster.

The 5S-state model employed in this study has provided further insights into the effects of external proteins on the heterogeneity of the oxygen evolution process. Specifically, the decrease in the *d* parameter observed in samples without external proteins suggests a diminished contribution of the fast oxygen release pathway. Remarkably, the removal of PsbQ and PsbP resulted in a nearly threefold reduction in the contribution of the fast pathway to O_2_ evolution. Furthermore, in PSII samples lacking all three external proteins (PsbQ, PsbP, and PsbO), the oxygen evolution process occurred almost exclusively through the slow pathway, represented by the S4’ state in the model.

### 4.2. Kinetics of the Fast and Slow O_2_ Release Pathways

Oxygen release times obtained using the fast polarographic method are typically in the range of 0.8 ms to 4 ms, depending on the measurement technique and conditions, type of the sample, and how the O_2_ release times are determined [190,191,192,193,194,195,196]. The lower limit of oxygen release, determined experimentally using EPR oximetry measurements and obtained via direct analysis of the signal after the third flash from the fast electrode, is approximately 500 µs [133,197]. This limitation also results from the electron transition time between Q_A_ and Q_B_, which varies from about 0.2 ms to 0.8 ms. It depends on the redox state of Q_B_ and the number of positive charges accumulated in the OEC [198,199,200,201,202]. It should be noted that regardless of the state of the acceptor side, at the start of OEC operation after the dark adaptation period, in order to achieve O_2_ release in the four-cycle action of each reaction center, the reduced and protonated quinone at the Q_B_ site must be replaced at least once by the oxidized quinone from the available external PQ pool. If the external pool of quinones was sufficient and the observed specific PQ diffusion pathway between the membrane and the Q_B_-binding pocket would allow for the attachment of quinones [84,203], the acceptor side of PSII is not a bottleneck in the kinetics of the oxygen release process. An increase in misses can be expected if one takes into account the cyclic flow around PSII postulated by various research groups. Cytochrome b_559_ plays an important role in this flow, among other things by causing the reduction of P680^+^ through a network of Car and Chl cofactors (for review see: [204,205,206]). Cytochrome b_559_, reduced by the PSII acceptor site, has also been shown to be able to reduce TyrD and TyrZ as well as *S_i(i_*_=2,3)_ states [207,208]. Recently, it has been postulated that the microstates (Q_A_Q_B_)^2−^ can reduce the Y^+^*S*_3_ and Y^+^*S*_2_ states, and this is coupled to the deprotonation of the PQ bound at the Q_C_ site [209,210]. As a result, the efficiency of the transitions between the *S*_2_ → *S*_3_ and *S*_3_ → *S*_4_ states can be reduced due to Y*S*_2_ ↔ Y^+^*S*_1_ and Y*S*_3_ ↔ Y^+^*S*_2_. This should lead to an increased damping of the oscillations of O_2_ evolution. It could be hypothesized that the partial relaxation of the *S*_2_ and *S*_3_ states to lower states, affecting backward recombination involving oxidized P680 and/or the cyclic electron flow around PSII, are responsible for the biphasic effect of oxygen evolution that we observed. Back reactions are certainly responsible for the increase in misses. However, if the effects we observed were related to this mechanism, there would be a problem explaining why the slow phase of oxygen evolution accelerates as the miss values increase. In fact, the opposite effect would be expected. The effect of cyclic electron transfer around PSII on the observed two-phase oxygen evolution will certainly be investigated.

The approximately 10-fold slower time constant of the O_2_ signal observed here is comparable in magnitude to the oxygen release times observed for thylakoids isolated from *Synechocystis* sp. PCC6803 mutants: D1-V185N/T, D1-N181A/S and D1-D61N/A. In these mutants, the time after which the oxygen release signal reached a maximum varied between 20 and 33 ms at pH 6.5 [211,212,213]. It is important to note that these times are about 20 to 30 times longer than those obtained for the wild form of *Synechocystis* sp. PCC6803. Mutations of the hydrophobic D1-V185 and hydrophilic D1-N181 are located near water molecules situated between TyrZ and D1-D61 and disrupt the extensive hydrogen bonding network between water molecules in the Cl1 water chain [95]. D1-N181 interacts with the chloride ion via hydrogen bonding and is one of the closest residues for water W2 bound to Mn4 [27,213]. The residue D1-D61 is directly hydrogen-bonded to the W1 ligand of Mn4 and is thought to be involved in proton removal from the OEC to the lumen and/or water entry [51,91,100,114,214]. The D1-V185 residue, being about 3.7 Å from the Mn_4_CaO_5_ complex, faces the Cl1 channel, which interacts with TyrZ, D1-Asp170, D1-D61, and D2-K317. Therefore, together with the surrounding water cluster, it may be involved in tuning the efficient relaxation processes of H-bond networks and/or proteins in the vicinity of the OEC. In this way, it may influence the efficiency of electron transfer to TyrZ^+^ [212].

Both the theoretical approach to the analysis of the oxygen release sequence under the influence of short flashes, which takes into account the bifurcation of the path of the O_2_ release and the experimental approach, based on a time-dependent measurement of the increase in O_2_ yield under the influence of the third flash, allow us to conclude that the observed heterogeneity is due to the functioning of the OEC. The consistency of the observed changes in the proportions of fast and slow O_2_ release pathways for the different samples, control and PSII with external proteins removed allows us to relate the obtained times to the fast and slow release of the oxygen molecule and thus to the kinetics of the processes occurring on the donor side of PSII. The reduction in the contribution of the fast phase to oxygen release in the case of PSII BBY—P,Q indicates a destabilization of the proton network of water inside the O4 and O1 channels due to the destruction of their outputs towards the lumen. This indicates not only an impairment of proton transport through these channels but also a reduced control of water access to the Mn_4_CaO_5_ complex. The impairment of the proton uptake through these channels is also manifested by the hindered transitions between the *S*_0_ → *S*_1_, *S*_1_ → *S*_2_, and *S*_3_ → *S*_0_. The additional extraction of the PsbO protein had no further effect on the efficiency of the transitions between the *S_i_* → *S_i_*_+1_ states. Still, it did result in the disappearance of the fast oxygen release phase. This means the transition between the *S*_3_ and *S*_0_ states in PSII BBY—O,P,Q occurs via the metastable *S*_4_ state. In this case, the only functional channel is Cl1A, as mentioned above, although some modifications of its functionality cannot be excluded. The PsbO protein has a region containing a hydrogen-bonding network near Cl-1 ion and a conserved residue R262 near the lumenal side, which can interact with the Mn_4_CaO_5_ cluster through a D1-D61-mediated hydrogen-bonding network extending to the PSII core proteins [215]. So, the organizational modification of D1 and D2, as shown on the donor side of PSII due to the extraction of external proteins, particularly PsbO, is possible in several ways. The absence of this protein destabilizes the Mn_4_CaO_5_ complex, affecting not only the organization of its protein ligands but also the entire proton network, to which water molecules and amino acids from the first and further coordination spheres contribute. The critical role of the hydrogen bonding network on the lumenal side of PSII, not only in the immediate vicinity of the OEC, was discussed in [92]. Furthermore, donor CP43 conformational changes also need to be considered [36]. The observed slowing of the fast phase in the sample lacking the PsbP and PsbQ proteins compared to the control is not surprising, as one would expect that a disruption of the coordinated deprotonation with water binding would lead to a slowing of the O_2_ evolution. Disabling some channels and undoubtedly reducing their efficiency in both transport and gating contribute significantly to this effect. The structural change in the Mn_4_CaO_5_ complex must also be considered. In contrast, the acceleration of oxygen release via the slow pathway may seem surprising. However, assuming that water is mainly supplied to the Mn_4_CaO_5_ complex through the O1 channel, the lack of gating of the water flow as a result of modification of the channel is the simplest explanation for the observed effect. Such a mechanism to control water access to the O1 channel with D1-E329 has been proposed for cyanobacteria [98]. The above hypothesis is highly plausible if one assumes that water binding to the manganese complex is an equally important mechanism regulating OEC function in addition to proton extraction. This thesis is supported by the further significant acceleration of oxygen release in PSII BBY—O,P,Q when the Mn_4_CaO_5_ complex has open access to the aqueous environment, and the aqueous network could provide an effective channel for the removal of oxygen molecules. On the other hand, if the hydrophobic matrix is responsible for the removal of O_2_, this would imply that the penetration of the oxygen molecule into the lipid layer is enhanced in PSII BBY—O,P,Q.

However, another completely different scenario can be outlined to explain the observed changes in the contribution and rate of these two O_2_ release pathways due to depletion of external proteins. EPR measurements showed that both LS and HS *S*_2_ states exist in higher plants [215]. This is attributed to the coexistence of two different isomers of the Mn_4_CaO_5_ complex (see Section 4 above). The depletion of the two outer proteins, PsbQ and PsbP, led to a significant decrease in the ratio of the HS signal to the LS signal for the *S*_2_ state. The depletion of all three outer proteins, i.e., including the PsbO protein, resulted in a complete loss of the HS signal for this state [215]. These results provide evidence for the regulatory role of the external proteins in stabilizing the HS *S*_2_ state. Thus, the fast transition pathway from *S*_3_ to *S*_0_ could be associated with the HS *S*_2_ state and the slow pathway, which requires consideration of the metastable *S*_4_ state, with the LS *S*_2_ state. We do not know how the following stages of the Kok cycle proceed. However, this direct correlation of the two *S*_2_ spin states with the respective fractions of the two oxygen release pathways suggests that the bifurcation of the mechanism leading to O-O bond formation and O_2_ release occurs at an earlier stage than the final transition *S*_3_ → (*S*_4_/*S*_4_’) → *S*_0_.

However, the following examples, based on the study of oxygen evolution in the above-mentioned mutants, illustrate how difficult it is to identify the main cause that determines whether PSII will release oxygen at a fast or slow rate.

Although D1-D61A/N and D1-V185N mutants affect OEC functioning differently, both lead to the slowing of oxygen release. It has been, therefore, suggested that in these mutants, the Mn_4_CaO_5_ complex adopts a structural rearrangement and/or tautomerism that allows a similar mechanism of O-O bond formation and oxygen release [211]. The D1-V185 residue was shown to be involved in stabilizing the *S*_2_ state of the Mn_4_CaO_5_ complex in the LS state, and a significant slowing of O_2_ release (*t*_1/2_ ≈ 20 ms) was observed in the D1-V185T mutant, in which the HS *S*_2_ state was dominant [212]. However, in an independent experiment, an O_2_ release rate (*t*_1/2_ ≈ 1.5 ms) similar to the wild type was observed for this mutant, albeit with a reduced oxygen production efficiency of approximately 40% [216]. In contrast to the V185T mutant [212], the Mn_4_CaO_5_ complex in the V185N, D61A, and D61N mutants, for which slow O_2_ evolution was observed, gives rise to a multiband *S*_2_ state signal that is qualitatively similar to the LS *S*_2_ state favoured in wild-type cyanobacteria at pH ~6 [217,218,219].

From the results of experiments performed on these cyanobacteria mutants, it can be concluded that the open (LS) or closed (HS) states of the manganese complex in *S*_2_ are not closely related to either the fast or slow oxygen release mechanism. Understanding the reasons for the different oxygen release times observed in the D1-V185T mutant by different research groups may provide valuable information about the underlying mechanism governing the rate of this process. Moreover, for the *S*_2_ and *S*_3_ states, there are potentially more possible configurations if differences in the hydration of the Mn_4_CaO_5_ cluster are also taken into account [33,34]. Nevertheless, it is unlikely that differences in the OEC due to the conformational heterogeneity of the *S*_2_ and/or *S*_3_ states themselves can explain the observed biphasic oxygen release. Thus, based on the results obtained for the mutants mentioned above, one may suggest that disruption of the hydrogen bond network in the immediate vicinity of the Mn_4_CaO_5_ complex slows down the release of oxygen as a result of delayed proton and/or electron transfer. In contrast, studies of the D1-N181A/S mutants have led to the conclusion that proton transfer is not impaired in their case and that the significant slowdown in oxygen yield is due to delayed O-O bond formation [213]. Interestingly, the *S*_2_ state was observed in both the LS and HS states in these mutants. The authors suggested that the positions and dynamics of critical water molecules required for efficient O–O bond formation may be perturbed in these mutants. Similarly, in the case of the D1-V185 and D1-D61 mutants, it was proposed that the changes introduced could affect substrate water’s movement and possibly the cluster’s associated isomerization [214,216].

Thus, it is clear that many factors can determine whether the release of oxygen in PSII will be fast or slow. Identifying the sequence of events that make a system fast or slow is currently very difficult, especially as it is so far not even certain when and how the O-O bond is formed [for review, see [31,43,168,220]]. Some believe that the formation of a bond between two oxygen atoms is possible only after the accumulation of four positive charges, i.e., during the transition of the manganese complex to the *S*_4_ state [44,74,75,217,221], while others think that it can already happen during the formation of the *S*_3_ state, which, like the *S*_2_ state, shows heterogeneity [23,31,52,69,71,161,222,223,224,225,226,227,228]. Each of the possible configurations of the Mn_4_CaO_5_ complex at the various stages of its reorganization associated with the accumulation of positive charge, the attachment of further water molecules, and the release of protons is closely related to the valence changes in specific Mn ions, and consequently, to the magnetic properties of the entire manganese cluster [for review, see [31]]. Due to the very short lifetime of the *S*_4_ state, one can only try to determine the organization of the OEC by modeling. This means that it is not possible to state unequivocally what is the actual stage of O-O formation and O_2_ release. How the reorganization of the Mn_4_CaO_5_ cluster itself may occur during the Kok cycle has been the subject of many hypotheses [for review, see [30,34,35,75,187,229]]. Recent studies of the *S*_3_ → (*S*_4_) → *S*_0_ transition kinetics using microsecond Fourier transform infrared (FTIR) spectroscopy [41] and serial femtosecond X-ray crystallography snapshots [40] have allowed for the approximation of the timescale of subsequent steps in the multistep O_2_ formation process. At least a two-step deprotonation of Mn_4_CaO_5_ was observed in the stage before TyrZ^+^ reduction and after water binding refilling the vacant site created by O_2_ release. Both experiments showed that the appearance of the peroxide after about 1.2–2.5 ms is the intermediate and slowest step before the formation of the O_2_ molecule. Furthermore, in both works, the D1-D61 pathway (Cl1 channel) was identified as responsible for the exit of both protons from Mn_4_CaO_5_ and D65/D312 as a regulator of these deprotonations. The mechanism of Mn_4_CaO_5_ cluster reorganization and substrate water binding proposed by both groups is based on its open cubic structure. This structure is typically observed in cyanobacteria. In higher plants, high-spin *S*_2_ and *S*_3_ states are detected, which are often explained by the closed cubic structure of the Mn_4_CaO_5_ complex [35]. Consequently, there have also been theoretical proposals for a different pathway leading to O-O bond formation and O_2_ release than the one proposed, for example, in [41], which involves Ca/Mn3/Mn4 μ3-oxo (O5) and Mn1(IV)-oxyl (Ox^−^), or in [40], where different variants have been suggested, not excluding the possibility of the involvement of the Mn(V) state in the final transition step, i.e., in the *S*_4_ state, prior to water binding to the substrate. A ‘nucleophilic oxy-oxo coupling’ mechanism between Mn4(V) = oxo and μ3-oxo (O5) has been proposed for the final *S*_4_ state in the case of the closed cubic structure of the Mn_4_CaO_5_ complex [230]. However, even here, a second pathway for the open cubane structure in the *S*_4_ state leading to an identifiable final *S*_0_ state has not been ruled out. Nevertheless, each of the proposed scenarios of O-O bond formation and O_2_ release requires synchronized receipt of protons (H^+^) and O_2_ and water binding to restore the *S*_0_ state.

## 5. Conclusions

In summary, this study emphasizes the critical role of external proteins in promoting the fast oxygen release pathway, which contributes to the biphasicity observed in the overall oxygen evolution process. These findings underscore the importance of external proteins in regulating the efficiency and dynamics of water splitting within the OEC. Moreover, it is evident that the conformation of the *S_i_* states of the water oxidizing enzyme is not the primary factor influencing the rate of oxygen release in the studied systems. Independent experimental and theoretical evidence from other research groups further supports the notion that the O_2_ evolution process must be considered in the context of the mutual influence of structural changes in the Mn_4_CaO_5_ complex, its protonation states, bound water molecules, and the functioning of water channels. Numerous studies have demonstrated the impact of external proteins on the stabilization and activity of the Mn_4_CaO_5_ complex [65,122,231,232,233,234,235,236]. However, their role in PSII heterogeneity has not been explored until now. Furthermore, it is essential to recognize that the entire protein–lipid–dye matrix forming the PSII system significantly influences the oxygen release process from water. The overall reorganization capacity of the PSII system plays a critical role in this process, including the local dynamics of the hydrogen network near the OEC and the influence of external proteins, as well as the dynamics on the acceptor side [36,42,44,71]. While changes on the acceptor side may potentially account for some biphasicity in oxygen release, this explanation becomes less plausible in PSII samples lacking PsbQ, PsbP, or PsbO proteins. In these samples, it is difficult to attribute any acceleration of the slow oxygen release pathway to conformational changes in the Q_B_ binding site. Additionally, no significant changes in transfer probabilities between *S_i_* states were observed compared to samples depleted of only PsbQ and PsbP.

This research did not examine the impact of Cl^-^ ions on the heterogeneity of the O_2_ evolution process, as the systems were operated in an environment with adequate chlorine ion concentration. However, it is possible that the release of Cl^-^ ions could affect the observed results, given their potential influence on the Cl1 channel’s efficiency in accepting protons from the Mn_4_CaO_5_ complex and delivering water molecules to it [237,238,239,240].

A key aspect of this work is the use of a method that is independent of any OEC operating model and assumptions regarding the initial states of the acceptor and donor sides of PSII. This allows for a more unbiased investigation into the two-phase nature of oxygen evolution, ensuring that the findings are not influenced by preconceived notions or specific model parameters.

In conclusion, these studies emphasize the pivotal role of extrinsic proteins in controlling the efficiency of the fast pathway for oxygen evolution in photosystem II (PSII).

## Figures and Tables

**Table 1:** Transition parameters and the initial *S_i_*-state distribution estimated according to the 5S-state model [37] (see Equation (S3) and the discussion in Section 2) for the PSII BBY control sample and PSII BBY depleted of two or three external proteins. Theoretical data are presented in Figure 5 (red-filled circles).

Parameters	*α*_0_	*α*_1_	*α*_2_	*α*_3_	*d*	*S*_0_	*S*_1_	*S*_2_	*S*_3_	*C*	*pfq*
PSII BBY control	0.001	0.001	0.785	0.001	0.85	0.05	0.87	0.08	0.00	0.995	0.0054
PSII BBY—P,Q	0.55	0.28	0.78	0.12	0.30	0.04	0.88	0.08	0.00	0.967	0.0165
PSII BBY—O,P,Q	0.51	0.27	0.79	0.16	0.02	0.282	0.530	0.130	0.058	0.990	0.0139

Parameters: *α_i_*—the failure rate of the trapping centers (called misses); *d*—the contribution of the fast mode to the O_2_ yield; *S_i_*—the initial distribution of the manganese states in the OEC; *C*—the parameter describing the fraction of active photosystems (the quenching parameter due to not sufficient amount of quinone acceptors); *pfq*—the parameter of fit quality described in Supplementary Materials, Equation (S5).

**Table 2:** Fitted parameters *A_fast_*, *A_slow_*, *τ_fast_*, and *τ_slow_* related to the amplitudes and time constants characterizing the fast and slow mode of O_2_ release (using Equation (1)).

Parameters	*A_fast_*	*τ_fast_* [ms]	*A_slow_*	*τ_slow_* [ms]
PSII BBY control	0.73 ± 0.08	4.1 ± 1.8	0.27 ± 0.08	44.2 ± 14.7
PSII BBY—P,Q	0.26 ± 0.09	6.2 ± 3.6	0.74 ± 0.06	22.2 ± 4.4
PSII BBY—O,P,Q	0	-------	1.00 ± 0.03	13 ± 2

## Data Availability

The data that support the findings of this study are available from the corresponding author upon reasonable request.

## Supplementary Materials

The following supporting information can be downloaded at: https://www.mdpi.com/article/10.3390/cimb46070428/s1, Figure S1: Examples of raw data of oxygen evolution under short saturating flashes separated by 300 ms obtained for (a) PSII BBY control, (b) PSII BBY—P,Q and (c) PSII BBY—O,P,Q.; Figure S2. Reduction of active reaction centers due to elution of two (PSII BBY—P,Q) and three (PSII BBY—O,P,Q) external proteins compared to the PSII BBY control sample; Figure S3. Flash-induced oxygen yield pattern in intact PSII BBY (a) and PSII BBY depleted of the extrinsic proteins by NaCl (b) and MgCl_2_ (c) washing (…) The red and blue symbols represent the theoretical fits of the corresponding experimental data, which were obtained using homogeneous Kok models with equal misses and with equal misses and double hits, respectively; Figure S4. Flash-induced oxygen yield pattern in control PSII BBY (A) and PSII BBY depleted of the extrinsic proteins by NaCl (B) and MgCl_2_ (C) washing. (…) In all cases, the black squares represent experimental data. The green, light blue, red, and dark blue symbols represent data simulated using the heterogeneous 5S-state extended Kok model for the parameters given in Table 1 (main text) for cyclic changes in the parameters *α_i_* when the largest missing parameter is set to *α*_0_, *α*_1_, *α*_2_ and *α*_3_, respectively, as indicated in the legends of the following figures: Figure S5. The example polarographic signals of oxygen evolution under the 3rd flash of light for the control PSII BBY (black symbols), PSII BBY—P,Q (magenta symbols) and PSII BBY—O,P,Q (blue symbols): (a) raw data and (b) normalized, smoothed signals shifted to 0 on the time scale; Figure S6. SDS-PAGE electrophoretogram of PSII BBY isolated from tobacco: Contr—control, NaCl—the sample depleted of PsbQ (17 kDa) and PsbP (23 kDa) proteins (PSII BBY—P,Q), MgCl_2_—the sample depleted of PsbQ (17 kDa), PsbP (23 kDa) and PsbO (33k Da) proteins (PSII BBY—O,P,Q), M—protein ladder; Figure S7: The differences between the experimental points and the theoretical curve when the data presented in Figure 6 (main text) were fitted freely without any assumptions (a) for PSII BBY—P,Q and (b) for PSII BBY—O,P,Q). The difference spectrum between the experimental data and the theoretical curve, assuming the time constants obtained for the PSII BBY_control_ sample (c) for PSII BBY—P,Q and (d) for PSII BBY—O,P,Q). The deviations of the fixed parameter curve from the free fit curve are shown by the blue line in (c) and (d).(…); Table S1. Transition parameters and the initial *S_i_*-state distribution estimated according to the standard Kok model assuming equal misses (red filled or open circles in Figure S3) or equal misses and double hits (blue filled circles in Figure S3) for the PSII BBY control sample and PSII BBY depleted of two or three external proteins. Refs. [241,242,243,244,245,246] are cited in the Supplementary Materials.
