# Supplementary material for: The Effect of Removal of External Proteins PsbO, PsbP and PsbQ on Flash-Induced Molecular Oxygen Evolution and Its Biphasicity in Tobacco PSII
Source: Curr Issues Mol Biol. 2024 Jul 8;46(7):7187–218. doi: 10.3390/cimb46070428 (PMC11276211; doi:10.3390/cimb46070428)
Supplement: Supplementary file 1 [file cimb-46-00428-s001.zip › cimb-3064163-latest update.pdf]

Supplementary Materials

The effect of removal of external proteins PsbO, PsbP and PsbQ on flash-induced molecular oxygen evolution and its biphasicity in tobacco PSII

Sonia Krysiak, Kvetoslava Burda*

Faculty of Physics and Applied Computer Science, AGH University of Krakow, al. Mickiewicza 30, 30-059 Krakow, Poland

Corresponding author: kvetoslava.burda@fis.agh.edu.pl

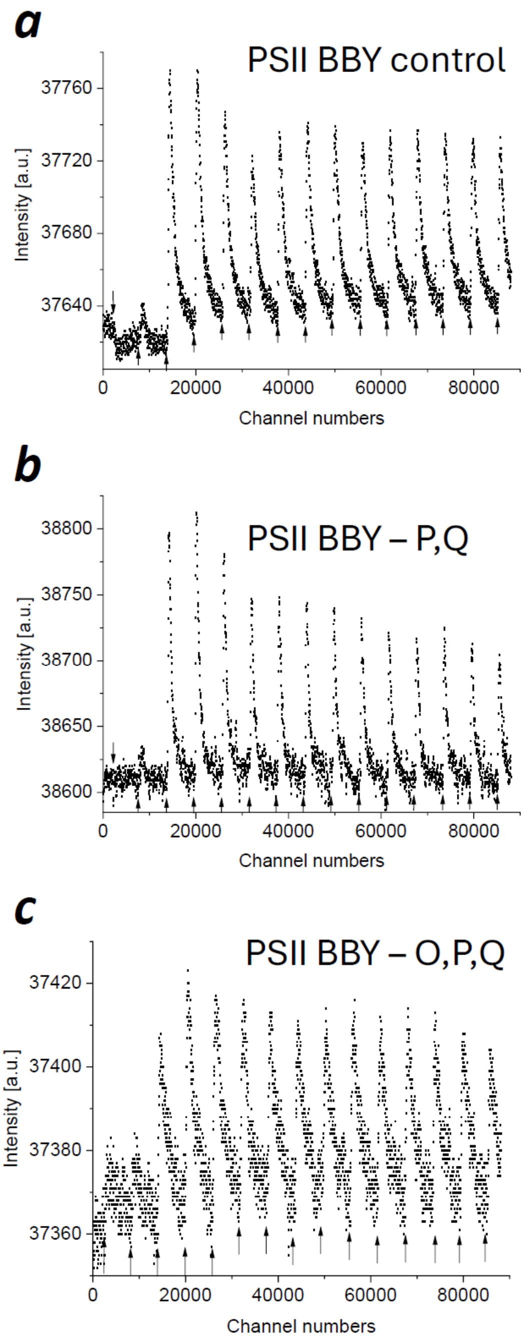

Figure 1S Examples of raw data of oxygen evolution under short saturating flashes separated by 300 ms, obtained for (a) PSII BBY control, (b) PSII BBY - P,Q and (c) PSII BBY - O,P,Q. The samples were suspended in Hepes I buffer at pH 6.5.

Due to the large number of experimental points, it is only possible to display the data in compressed form here.

In the presented data, blue arrows signify the points at which short, saturated flashes of light were applied during the measurement process. The initial flash occurred 100 ms after the start of the measurement, with subsequent flashes administered at regular intervals of 300 ms throughout the duration of the experiment.

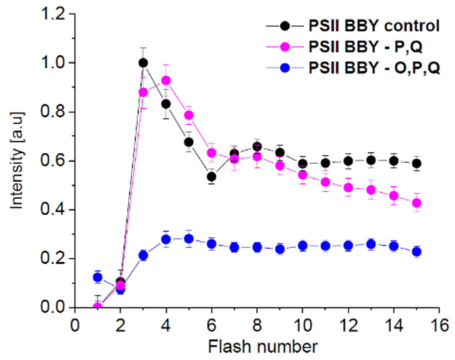

Figure 2S Reduction of active reaction centres due to elution of two (PSII BBY - P,Q) and three (PSII BBY - O,P,Q) external proteins compared to the PSII BBY control sample. The experimental data are the mean values obtained from 3 independent measurements in each case. The error bars correspond to the maximum error. Flash-induced oxygen yield patterns were obtained under the same experimental conditions for samples suspended in the Hepes I buffer, pH 6.5. The separation period between flashes was 300 ms.

The common problem of observing an increasing number of double hits with increasing number of misses indicated in [139,140] has also puzzled other researchers who have tried to explain this problem in terms of the functioning of the PSII complex, still assuming a model with equal misses and equal double hits by introducing additional parameter related to the backward-transitions or deactivation of the reaction centres (see for example [138,241-243]) or even introducing asymmetry to Markov model (parameter of asymmetry) [244]. In addition, to improve the fit of the observed O_2 evolution processes, the formal introduction of the S_{-1} state has been proposed, even in the absence of exogenous reducing agents (for example: [243,245]). There are many more complicated models which, because of the large number of parameters, require introducing predetermined values for some of the parameters, which in turn affect the other parameters to be fitted (see for example: [143,246]). Some parameters specified in the model (e.g. equal or unequal double hits) are omitted.

The theoretical fit of our experimental data obtained using homogeneous Kok models with equal misses (transition matrix - Eq. S1) or equal misses and double hits (transition matrix - Eq. S2) is shown below (Figure S3). The resulting parameters are given in Table S1.

$$\boldsymbol{A}\cdot\overrightarrow{\boldsymbol{S^n}}=\overrightarrow{\boldsymbol{S^{n+1}}}=\begin{bmatrix}\alpha & 0 & 0 & \beta\\ \beta & \alpha & 0 & 0\\ 0 & \beta & \alpha & 0\\ 0 & 0 & \beta & \alpha\end{bmatrix}\cdot\begin{bmatrix}S_0^n\\ S_1^n\\ S_2^n\\ S_3^n\end{bmatrix}=\begin{bmatrix}S_0^{n+1}\\ S_1^{n+1}\\ S_2^{n+1}\\ S_3^{n+1}\end{bmatrix} \quad \text{(S1)}$$

where $\alpha + \beta = 1$

$$\boldsymbol{G}\cdot\overrightarrow{\boldsymbol{S^n}}=\overrightarrow{\boldsymbol{S^{n+1}}}=\begin{bmatrix}\alpha & 0 & \gamma & \beta\\ \beta & \alpha & 0 & \gamma\\ \gamma & \beta & \alpha & 0\\ 0 & \gamma & \beta & \alpha\end{bmatrix}\cdot\begin{bmatrix}S_0^n\\ S_1^n\\ S_2^n\\ S_3^n\end{bmatrix}=\begin{bmatrix}S_0^{n+1}\\ S_1^{n+1}\\ S_2^{n+1}\\ S_3^{n+1}\end{bmatrix} \quad \text{(S2)}$$

where $\alpha + \beta + \gamma = 1$

In both cases: n – flash number and $\sum_{i=0}^{3} S_i = 1$.

Oxygen evolution under the nth - flash is given by:

$Y^n = \beta_3 S_3^{n-1}$ for the model described by eq. S1

or

$Y^n = \beta S_3^{n-1} + \gamma S_2^{n-1}$ for the model described by eq. S2.

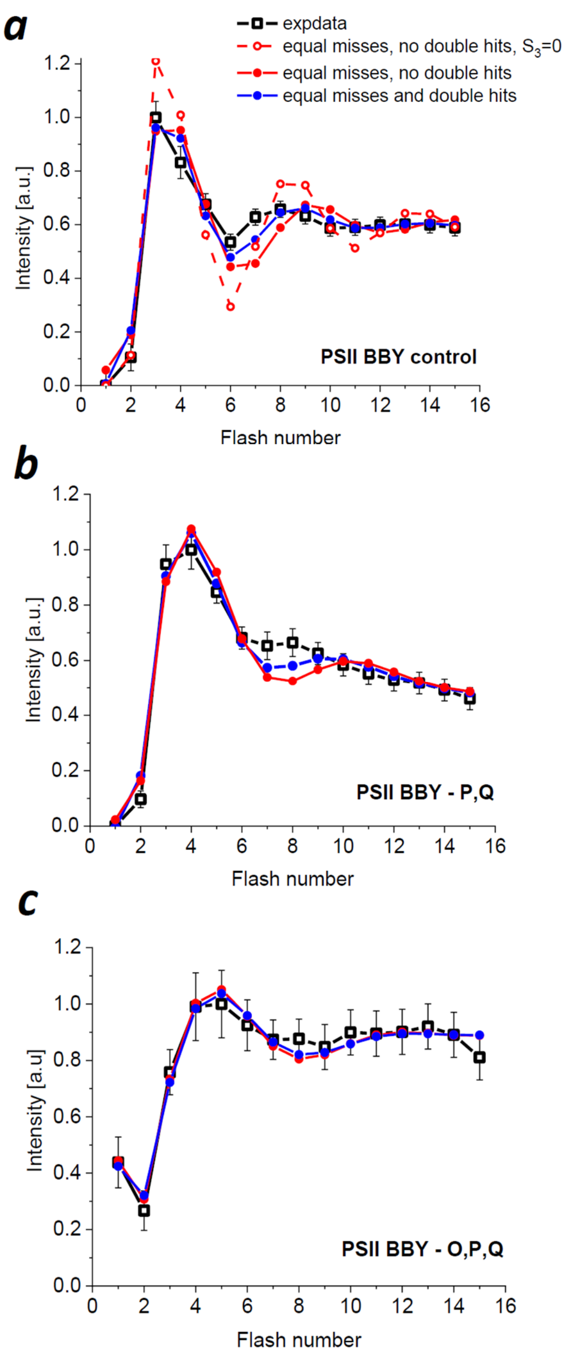

Figure S3. Flash-induced oxygen yield pattern in intact PSII BBY (a) and PSII BBY depleted of the extrinsic proteins by NaCl (b) and $MgCl_2$ (c) washing. Samples were suspended in the Hepes I buffer pH 6.5. The flashes were 300 ms apart. The experimental data are always normalised to the maximum amplitude of the O_2 evolution. The experimental data are the mean values obtained from 3 independent measurements in each case. The error bars correspond to the maximum error.

In all cases the black squares represent experimental data. The red and blue symbols represent the theoretical fits of the corresponding experimental data, which were obtained using homogeneous Kok models with equal misses and with equal misses and double hits, respectively.

Table S1. Transition parameters and the initial S_i-state distribution estimated according to the standard Kok model assuming equal misses (red filled or open circles in Figure S3) or equal misses and double hits (blue filled circles in Figure S3) for the PSII BBY control sample and PSII BBY depleted of two or three external proteins.

Parameters	α	γ	S_0	S_1	S_2	S_3	C	*pfq*
equal misses and no double hits								
PSII BBY control	0.329	0	0.105	0.765	0.106	0.023	0.999	0.078
	0,25	0	0.12	0.82	0.06	0.000*	0.996	0.083
PSII BBY – P,Q	0.431	0	0.097	0.805	0.091	0.007	0.969	0.059
PSII BBY - O,P,Q	0.450	0	0.276	0.534	0.058	0.133	0.995	0.021
equal misses and double hits								
PSII BBY control	0.302	0.052	0.175	0.822	0.003	0.000	0.995	0.034
PSII BBY – P,Q	0.399	0.038	0.196	0.804	0.000	0.000	0.963	0.031
PSII BBY - O,P,Q	0.460	0.012	0.289	0.536	0.048	0.127	0.990	0.019

Parameters: α – the failure rate of the trapping centres (called misses), γ – fraction of doubly excited centres (called double hits), S_i – the initial distribution of the manganese states in the OEC, C – the parameter describing the fraction of active photosystems (the quenching parameter due to not sufficient amount of quinone acceptors); *pfq* – the parameter of fit quality defined in eq. S5

* The initial value of S_3 has been set to 0.

The data of oxygen evolution oscillations under the influence of short flashes of saturating light presented in the main work were fitted using the 5S-state extended Kok model [37]. The transition matrix $\boldsymbol{B}$ is given below:

$$\boldsymbol{B}\cdot\overrightarrow{\boldsymbol{S^n}}=\overrightarrow{\boldsymbol{S^{n+1}}}=\begin{bmatrix}\alpha_0 & 0 & 0 & \beta_3 d & 0\\ \beta_0 & \alpha_1 & 0 & 0 & 1\\ 0 & \beta_1 & \alpha_2 & 0 & 0\\ 0 & 0 & \beta_2 & \alpha_3 & 0\\ 0 & 0 & 0 & \beta_3(1-d) & 0\end{bmatrix}\cdot\begin{bmatrix}S_0^n\\ S_1^n\\ S_2^n\\ S_3^n\\ S_4^n\end{bmatrix}=\begin{bmatrix}S_0^{n+1}\\ S_1^{n+1}\\ S_2^{n+1}\\ S_3^{n+1}\\ S_4^{n+1}\end{bmatrix} \tag{S3}$$

where n – flash number, $\alpha_i+\beta_i=1$ for i=0, 1, 2, 3, $0\le d\le 1$ and $\sum_{i=0}^{4} S_i=1$.

Oxygen evolution under the nth-flash is given by:

$$Y^n = C(\beta_3 d S_3^{n-1} + \beta_3(1-d)S_3^{n-1}) = C\beta_3 S_3^{n-1} \tag{S4}$$

The program written in C uses the standard minimization function for the following expression:

$$\Delta y^2 = \sum_{k=1}^{n}\left[Y_{\exp}^k - Y_{theo}^k\cdot\left(\frac{\sum_{k=1}^{n} Y_{\exp}^k}{\sum_{k=1}^{n} Y_{theo}^k}\right)\right]^2 \tag{S5}$$

where $Y_{\exp}^k$ and Y_{theo}^k are experimental and theoretical data, respectively.

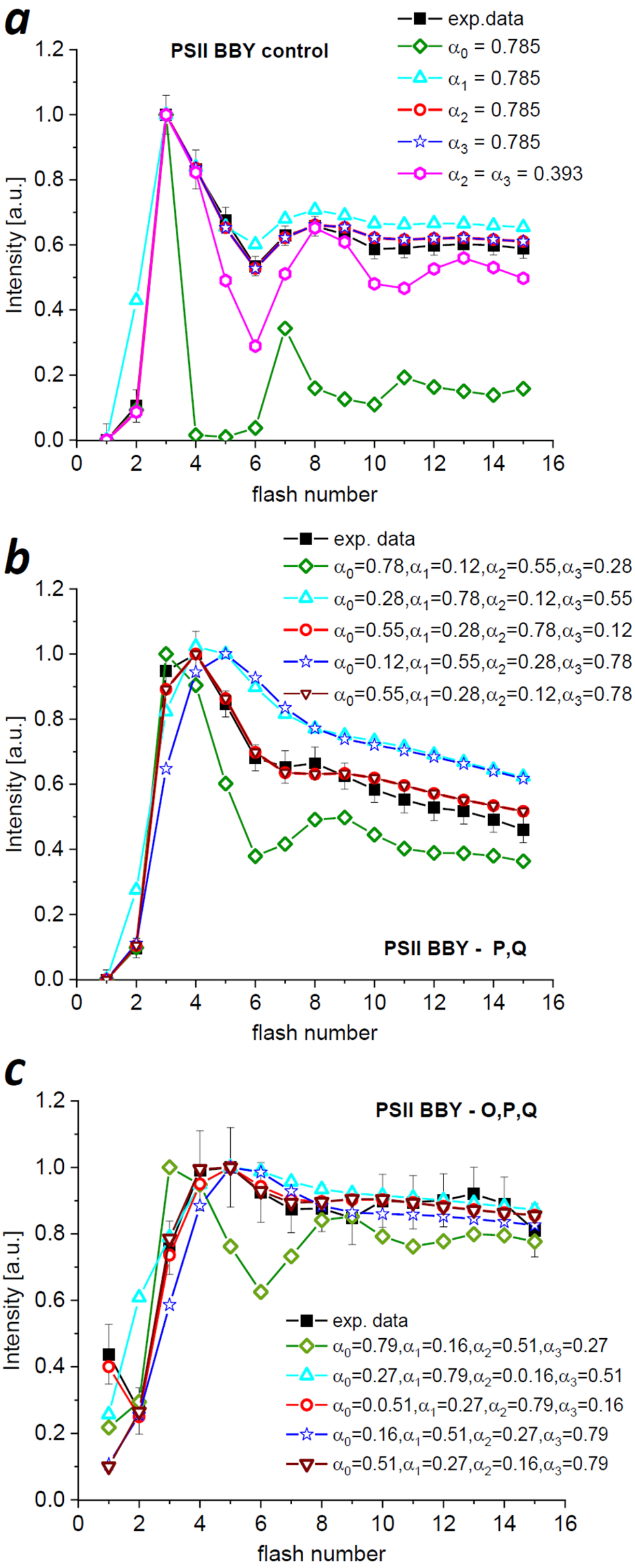

Figure S4. Flash-induced oxygen yield pattern in control PSII BBY (a) and PSII BBY depleted of the extrinsic proteins by NaCl (b) and $MgCl_2$ (c) washing. Samples were suspended in the Hepes I buffer pH 6.5. The flashes were 300 ms apart. All data are normalised to the corresponding maximum O_2 release amplitude. The experimental data are the mean values obtained from 3 independent measurements in each case. The error bars correspond to the maximum error.

In all cases the black squares represent experimental data. The green, light blue, red and dark blue symbols represent data simulated using the heterogeneous 5S-state extended Kok model for the parameters given in Table 1 (main text) for cyclic changes of the parameters α_i when the largest missing parameter is set to α_0, α_1, α_2 and α_3, respectively, as indicated in the legends of the following figures. Additional simulated data are shown in Figures A, B and C. For the PSII BBY control sample, magenta symbols represent data simulated assuming $\alpha_2 = \alpha_3 = 0.393$, i.e. the half value of the maximum α_2 obtained from fitting the experimental data (Table 1). Figures B and C, for samples PSII BBY - P,Q and PSII BBY - O,P,Q respectively, show additional simulated data (vine triangles) for the case of exchanging only the parameters α_2 and α_3 given in Table 1.

We performed a series of simulations to illustrate how the pattern of oxygen evolution in the heterogenous 5S-state extended Kok model depends on α_i parameters. In particular, we checked how the pattern changes when the α_i values are swapped. The results are shown in Figure S4. They consistently demonstrate that the model is sensitive to alterations in the α_i parameters, with the sole exception being the symmetry between α_2 and α_3. However, the symmetry only holds when the initial occupation of state S_3 is equal to 0, and it is related to a symmetry of the eigenvalues of the matrix B (eq. S3) with respect to the exchange of α_2 and α_3. To further illustrate this point, we present in Figure 4Sa simulations for the control sample where $\alpha_2 = \alpha_3$ and are equal to half their summed values obtained from fitting. The oscillation pattern observed in this scenario deviates significantly from the pattern in which the values of α_2 and α_3 are swapped. Even with a small occupancy of the S_3 state, this symmetry is broken, and the miss parameter α_2 has the highest value. The difference between the oxygen evolution patterns increases with increasing occupancy of the S_3 state, as can be seen in the example of the PSII BBY - O,P,Q sample, where the occupancy of the S_3 state is almost 6%.

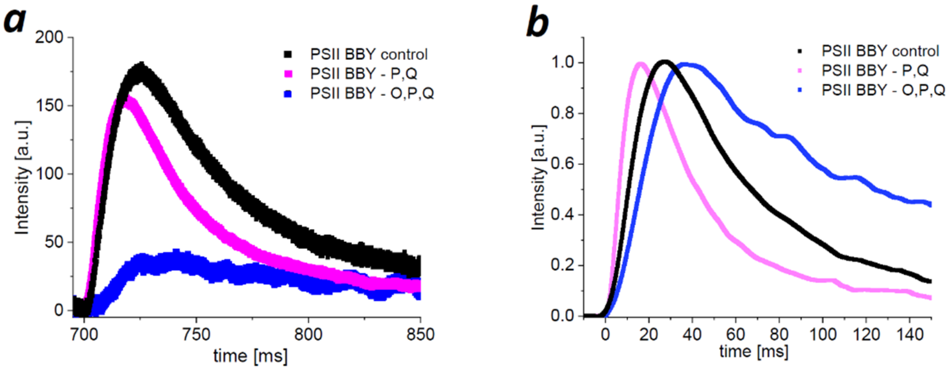

Figure S5. The example polarographic signals of oxygen evolution under the 3rd flash of light for the control PSII BBY (black symbols), PSII BBY – P,Q (magenta symbols) and PSII BBY – O,P,Q (blue symbols): (a) raw data and (b) normalised, smoothed signals shifted to 0 on the time scale. In (a) the signals start to rise from 700 ms. This is due to the start of the measurement 100 ms before the first flash and the sum of the 2 x 300 ms times resulting from the flash separation. The signals were obtained under the same experimental conditions for samples suspended in the Hepes I buffer, pH 6.5.
The red solid line ending with arrows shows how the amplitudes of oxygen evolution under the influence of short flashes were determined. The example is the signal obtained for the control sample presented in figure (a).
The maximum signal increase for the control sample, PSII BBY - P,Q and PSII BBY - O,P,Q was observed after approximately 26 ms, 16 ms and 36 ms respectively. Their signal half-times are about 10 ms, 6 ms and 16 ms respectively.

Based on the analysis of these signals, different trends in O_2 release rate were found in the tested samples compared to the times shown in Table 1. We suggest that the main factors responsible for the observed directions of change in the shape of the polarographic signal after removing external proteins are differences in the surface potential and/or packing density of the tested samples. These factors alter the thermodynamic equilibrium in the sample-electrode contact region. In our research, we are planning to investigate this issue.

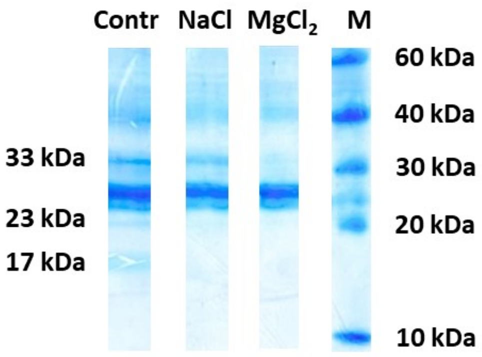

Figure 6S. SDS-PAGE electrophoretogram of PSII BBY isolated from tobacco: Contr – control, NaCl – the sample depleted of PsbQ (17 kDa) and PsbP (23 kDa) proteins (PSII BBY – P,Q) , $MgCl_2$ – the sample depleted of PsbQ (17 kDa), PsbP (23 kDa) and PsbO (33k Da) proteins (PSII BBY – O,P,Q), M – protein ladder.

The locally visible irregular light or dark wrinkles come from the folds of the film used to protect the gel from drying out.

Even if a small fraction of the PSII samples exposed to high concentrations of NaCl or $MgCl_2$ had still contained external proteins, this would not have had a statistically significant effect on the observed results or on the conclusions drawn from the experiments. The changes in oxygen evolution observed in the samples analysed are significant and consistent with the expected/known behavioural trends of photosystem II (PSII) lacking external proteins.

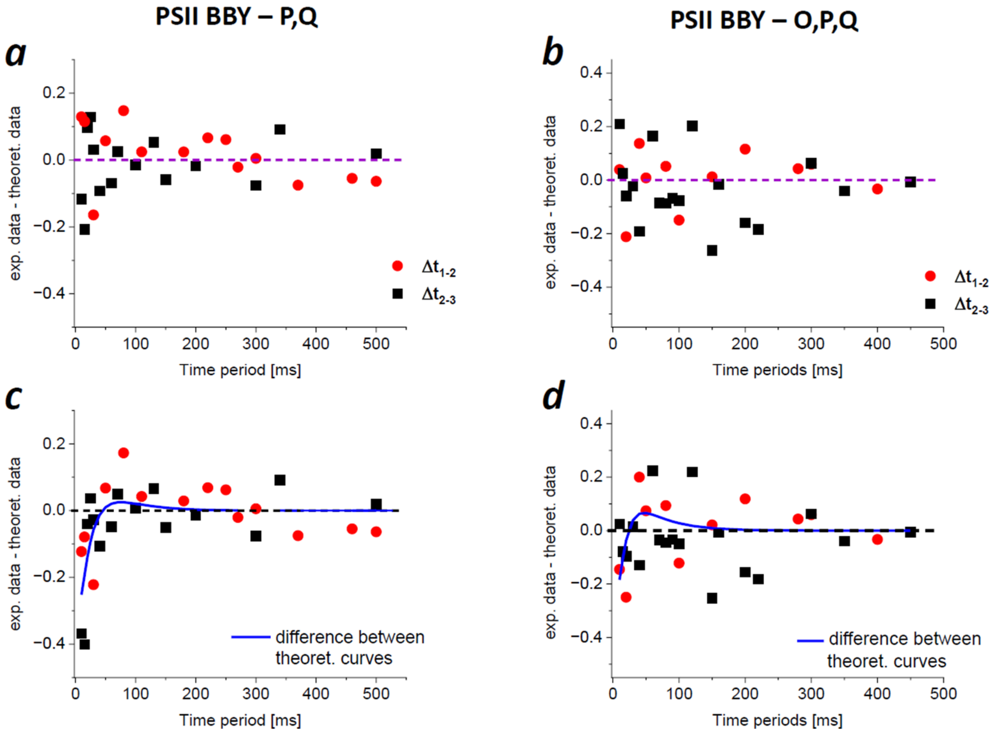

Figure S7. The differences between the experimental points and the theoretical curve when the data presented in Fig.5 (main text) were fitted freely without any assumptions (a) for PSII BBY - P,Q and (b) for PSII BBY - O,P,Q). The difference spectrum between the experimental data and the theoretical curve, assuming the time constants obtained for the PSII BBY$_{control}$ sample (c) for PSII BBY - P,Q and (d) for PSII BBY - O,P,Q). The deviations of the fixed parameter curve from the free fit curve are shown by the blue line in (c) and (d). Thus this blue line shows the mismatch of experimental points assuming a model with fixed time constants as for the control sample For clarity, we have omitted the errors.

As can be seen in Fig. S7 c and d, for short Δt_{1-2} and of Δt_{2-3} (< about 40 ms) the experimental points are below the fixed time constant curve while for the range from about 40 ms to about 150 ms they are

slightly above. Therefore the blue lines deviate from zero within this ranges which indicates that fitting with τ_{fast} and τ_{slow} as in the control sample (see Table 2 in the main text) is not capable to reproduce the behaviour of the signal in this range correctly. This shows that the model with the fixed time constant, as obtained for the control, does not fit the data well. In addition, the parameter pfq is also higher for the fixed parameter model. In the case of PSII BBY – P,Q it is about 0.223 and 0.467 for fitting with free parameters and fixed time constants, respectively. In the case of PSII BBY – O,P,Q it is about 0.398 and 0.449 for fitting with free parameters and fixed time constants, respectively.
